# Testosterone Modulation of Muscle Transcriptomic Profile During Lifestyle Therapy in Older Men with Obesity and Hypogonadism

**DOI:** 10.1002/jcsm.13697

**Published:** 2025-01-27

**Authors:** Viola Viola, Tagari Samanta, Maria Liza Duremdes Nava, Alessandra Celli, Reina Armamento‐Villareal, Ngoc Ho Lam Nguyen, Georgia Colleluori, Yoann Barnouin, Nicola Napoli, Clifford Qualls, Benny Abraham Kaipparettu, Dennis T. Villareal

**Affiliations:** ^1^ Center for Translational Research on Inflammatory Diseases Michael E DeBakey VA Medical Center Houston Texas USA; ^2^ Division of Endocrinology, Diabetes and Metabolism Baylor College of Medicine Houston Texas USA; ^3^ Operative Research Unit of Osteo‐Metabolic and Thyroid Diseases Fondazione Policlinico Universitario Campus Bio‐Medico Rome Italy; ^4^ Department of Molecular and Human Genetics Baylor College of Medicine Houston Texas USA; ^5^ Division of Bone and Mineral Diseases Washington University in St Louis St. Louis Missouri USA; ^6^ Department of Mathematics and Statistics University of New Mexico Albuquerque New Mexico USA

**Keywords:** bone, lifestyle therapy, skeletal muscle, testosterone

## Abstract

**Background:**

Testosterone replacement therapy (TRT) added to lifestyle therapy can mitigate weight‐loss–induced reduction of muscle mass and bone mineral density (BMD) in older men with obesity and hypogonadism.

**Objective:**

To investigate the molecular mechanisms underlying the attenuation of muscle and BMD loss in response to TRT during intensive lifestyle intervention in this high‐risk older population.

**Methods:**

Among 83 older (≥ 65 years) men with obesity (BMI ≥ 30 kg/m^2^) and hypogonadism (early AM testosterone persistently < 300 ng/dL) associated with frailty (Modified Physical Performance Test score ≤ 31) randomized into 26‐week lifestyle therapy plus testosterone (LT+TRT) or placebo (LT+Pbo) in the LITROS trial, 38 underwent serial muscle biopsies for the muscle transcriptomics substudy.

**Results:**

Despite similar ~10% weight loss, lean body mass and thigh muscle volume decreased less in LT+TRT than LT+Pbo (−2% vs. −4%, respectively; *p* = 0.04). Hip BMD was preserved in LT+TRT compared with LT+Pbo (0.4% vs. −1.3%; *p* = 0.03). Muscle strength increased similarly in LT+TRT and LT+Pbo (23% vs. 24%; *p* = 0.95). Total testosterone increased more in LT+TRT than LT+Pbo (133% vs. 32%; *p* = 0.005). Based on Next Generation Sequencing, of the 39 160 and 39 115 genes detected in LT+TRT and LT+Pbo, respectively, 195 were differentially expressed in LT+TRT and 158 in LT+Pbo. Gene Ontology enrichment analyses revealed that in LT+TRT, just four muscle‐related pathways (muscle organ development, muscle organ morphogenesis, regulation of skeletal muscle contraction, muscle atrophy) were downregulated and one pathway (muscle system process) was upregulated. In contrast, in LT+Pbo, nine muscle‐related pathways (muscle system process, muscle tissue development, muscle organ development, skeletal muscle tissue development, skeletal muscle organ development, skeletal muscle cell differentiation, muscle organ morphogenesis, response to stimuli involved in regulation of muscle adaptation, muscle atrophy) and one pathway related to bone (bone mineralization involved in bone maturation) were downregulated. Muscle system process was upregulated in LT+TRT but downregulated in LT+Pbo. RT‐PCR analyses showed that LT+TRT resulted in a higher expression of MYOD1 (*p* = 0.02) and WNT4 (*p* = 0.02), key genes involved in muscle and bone metabolism, respectively, compared with LT+Pbo. We also observed significantly higher mRNA expression of MYBPH (*p* = 0.006), SCN3B (p = 0.02) and DSC2 (*p* = 0.01), genes involved in the muscle system process, in response to LT+TRT compared with LT+Pbo.

**Conclusion:**

The addition of TRT to lifestyle therapy mitigates the weight‐loss–induced reduction of muscle mass and BMD via countering the weight‐loss–induced downregulation of genes involved in muscle and bone anabolism.

## 1 Introduction

Obesity, a multifactorial epidemic disease defined by a body mass index (BMI) of ≥ 30 kg/m^2^, is well‐known to exacerbate the age‐related decline in physical function leading to frailty, sarcopenia, and risk for falls and fractures [1, 2, 3, 4]. Among the numerous etiological factors contributing to the obesity‐ and age‐related decline in musculoskeletal integrity, decreased muscle and bone mass can be partially attributed to the intrinsic reduction of anabolic hormones such as testosterone [5]. In muscle physiology, testosterone is widely considered one of the primary drivers of muscle hypertrophy and concomitant gains in strength in men [6]. Moreover, the conversion of testosterone into oestrogen plays a crucial role not only in muscle development but also in regulating bone health in older men, including bone mineral density (BMD) [7]. The decline in endogenous testosterone levels typically arises in the fourth decade of life in men [8, 9] and may be further exacerbated by the simultaneous presence of obesity [10]. Recent clinical evidence suggests that obesity is a paramount contributory factor to the emergence of secondary hypogonadism in the male population [11, 12]. In older men, testosterone deficiency is closely linked to visceral adipose tissue dysfunction [13]. The increase in BMI leads to a decrease in testosterone levels in men [10], which in conjunction with the age‐related decline in anabolic hormones may contribute to the increased development of sarcopenia in older adults [2].

Lifestyle therapy (LT) consisting of weight loss and exercise training is the standard treatment for obesity [1]. However, weight loss induced by calorie restriction (CR) has been shown to decrease muscle and bone mass, which is further intensified by the natural loss that occurs with aging [2]. Our team has already demonstrated that the best strategy to improve functional status and ameliorate frailty in older adults with obesity consists of ~10% diet‐induced weight loss in combination with aerobic and resistance exercise [14, 15]. We showed that LT plays an important role in mitigating the weight‐loss‐induced reduction of muscle and bone mass by improving muscle protein synthesis and myocellular quality [16] and reducing bone turnover [17]. Given the high risk of hypogonadism in older adults with obesity [11, 12] alongside the improvements in frailty resulting from the LT [14, 15], we conducted a randomized controlled trial, the Lifestyle Intervention and Testosterone Replacement in Obese Seniors (LITROS Clinical Trials.gov, NCT02367105) to investigate whether testosterone replacement therapy (TRT) would augment the effects of LT on the musculoskeletal health of this high‐risk population [18]. In this regard, our LITROS trial showed that TRT added to LT further ameliorates the weight loss‐induced reduction of muscle mass and hip BMD in older men with obesity and hypogonadism [18]. However, the mechanisms behind the intricate interplay between TRT and LT and skeletal muscle and bone metabolism are still unknown. Therefore, the objective of the current study, the LITROS muscle transcriptomic substudy, was to investigate the molecular mechanisms underlying the protective effects of TRT on muscle and bone mass during LT of older men with obesity and hypogonadism. We hypothesized that TRT added to LT mitigates the weight loss‐induced reduction of muscle and BMD by positively modulating the skeletal muscle transcriptomic profile of frail older men with obesity and hypogonadism.

## 2 Methods

### 2.1 Study Design and Participants

**FIGURE 1:**
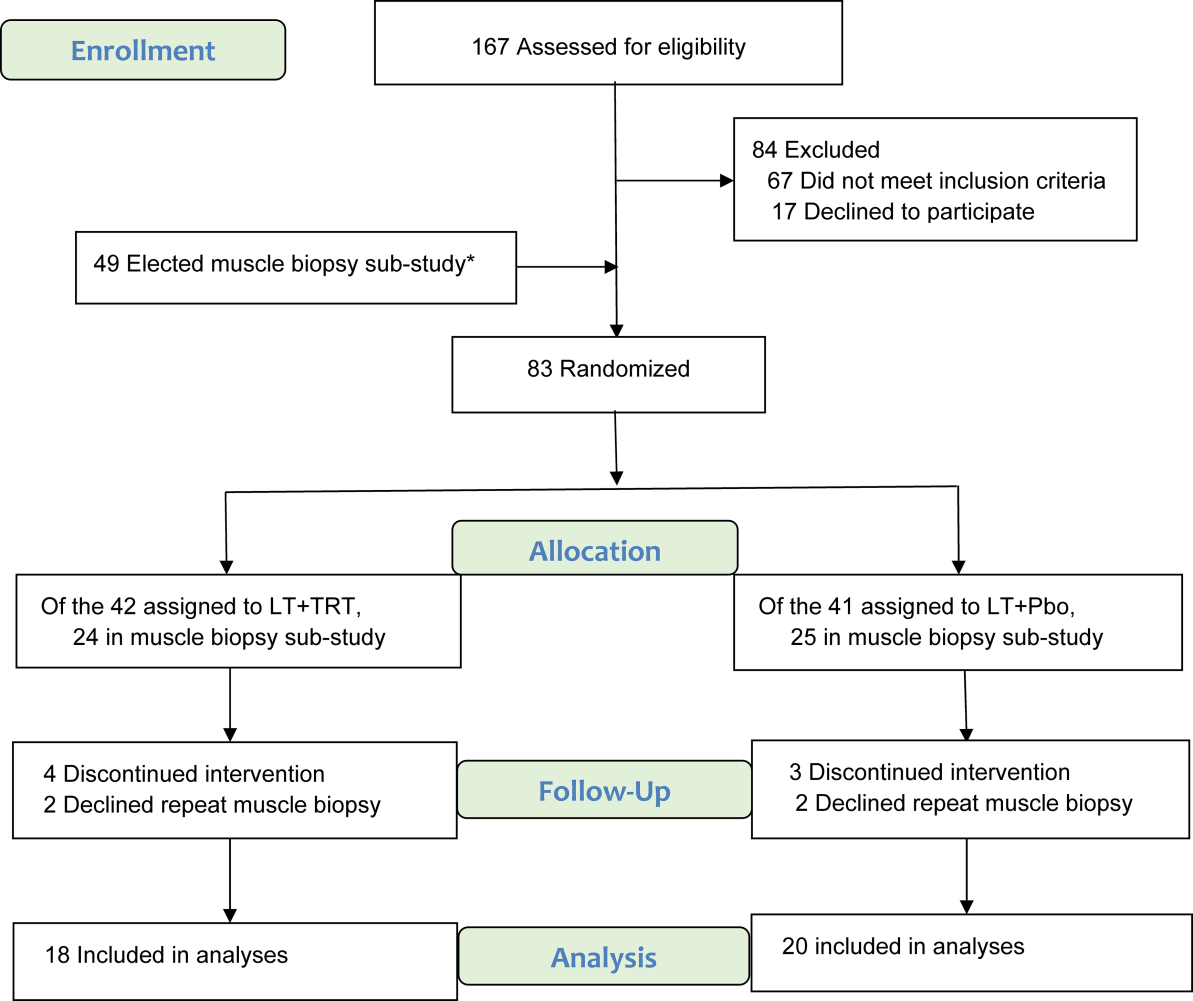
Screening, randomization, and follow‐up. Groups: lifestyle therapy (weight management and exercise training) plus placebo (LT+Pbo) and lifestyle therapy plus testosterone (LT+TRT). Note: *49 met inclusion criteria and elected muscle biopsy sub‐study and as expected, these were equally assigned to the study arms (LT+TRT vs LT+Pbo) by the subsequent randomization

Thirty‐eight individuals enrolled in the LITROS trial [18], who met the trial's criteria and agreed to undergo muscle tissue biopsies, were included in the muscle transcriptomic substudy. This substudy involved analyses of Next Generation Sequencing (NGS) before and after 26 week of intervention. Participants for the substudy were recruited prior to randomization in the main LITROS trial. Further details regarding the recruitment and randomization process are provided in Figure 1. The study was conducted at the Michael E DeBakey Veterans Affairs Medical Center (MEDVAMC) in Houston, Texas, and approved by the Institutional Review Board of Baylor College of Medicine and the Research and Development Committee of the MEDVAMC. Volunteers were recruited using advertisements and reviews of medical records at the MEDVAMC. Potential participants gave informed consent and underwent a comprehensive medical screening procedure, including a graded treadmill exercise stress test. Inclusion criteria were: veterans with older age (age ≥ 65 years), obesity (BMI ≥ 30 kg/m^2^), hypogonadism, (fasting early AM testosterone levels < 10.4 nmol/L on two separate mornings), mild‐to‐moderate frailty (Physical Performance Test (PPT) score of 18–31) [19], sedentary lifestyle (regular exercise <1 h per week), stable body weight (±2 kg) and stable medications for 6 months before enrolment. Exclusion criteria included: severe cardiopulmonary disease, musculoskeletal or neuromuscular impairments that precluded exercise training, use of bone‐acting drugs, history of prostate cancer, venous thromboembolism, untreated sleep apnoea, haematocrit > 50%, prostate findings of palpable nodule, prostate‐specific antigen ≥ 4 ng/mL and International Prostate Symptom score > 19.

After baseline testing, participants were randomly assigned, with stratification according to BMI < 35 kg/m^2^ or ≥ 35 kg/m^2^ into two groups: (i) lifestyle therapy plus TRT (LT+TRT) or (ii) lifestyle therapy plus placebo (LT+Pbo) for 26 weeks. Lifestyle therapy consisted of weight management and exercise training as previously described [18]. Briefly, participants were prescribed an energy deficit diet of 500 to 750 kcal/day from daily energy requirement and asked to meet weekly as a group for adjustment of calorie intake and behavioural therapy with a dietitian. The goal was to achieve weight loss of ~10% in 6 months. The exercise training consisted of combined aerobic and resistance training sessions thrice weekly at our facility as supervised by exercise trainers. For the TRT, Androgel 1.62% (Abbvie) was applied topically once daily in the morning. The placebo gel was formulated to have identical appearance as the active gel. The initial dose was 40.5 mg daily, which was expected to increase the testosterone levels to within the normal range for young men (19–40 years old). Testosterone level was measured 2 weeks after starting the intervention. If the testosterone level was not in the target range, an unblinded physician adjusted the dose and the measurement repeated after another two weeks. To maintain blinding, the dose was changed simultaneously in a participant receiving placebo.

Further details regarding the interventions including adherence data, exercise adaptations, and adverse events have been previously described [18].

### 2.2 Physical Function

Frailty was assessed using the Modified Physical Performance Test (PPT) which consists of nine tasks: six timed tasks (climbing one flight of stairs, standing up from a chair, walking 50 ft, putting on and removing a coat, picking up a penny, lifting a 7 lb book to a shelf) and other three tasks include going up and down four flights of stairs, making a 360° turn and, performing a progressive Romberg test. The score for each task ranges from 0 to 4; the perfect score is 36 [19]. Muscle strength was evaluated by measuring total 1‐repetition maximum (1‐RM), the total of the maximum weight the participant lifted, in one attempt, in the biceps curl, bench press, seated row, knee extension, knee flexion, and leg press.

### 2.3 Lean Body Mass, Muscle Volume, and Bone Mineral Density

Total lean body mass and BMD of the total hip were measured using dual‐energy X‐ray absorptiometry (Hologic and Horizon APEX Software 5.5.2), as described previously [18, 20]. Thigh muscle volume was measured using Magnetom Avanto (Siemens) scanner and analysed using Analyze Direct software (version 10.0; Mayo Clinic, Rochester, MN), as described elsewhere [18, 21].

### 2.4 Specimen Preparation

Muscle tissue samples were obtained at baseline and after six months under local anaesthesia using Tilley‐Henkel forceps. Lidocaine (2%) was injected at the biopsy site, and a small incision (~1 cm) was made to facilitate the passage of forceps (5 mm) into the vastus lateralis, from which approximately 150 mg of tissue was collected. Following collection, muscle specimens were meticulously cleared of any blood and adipose tissue, and then immediately snap‐frozen in liquid nitrogen for subsequent biochemical analysis of gene expression. Blood samples were drawn in the morning following an overnight fast. Total testosterone levels were quantified using liquid chromatography–tandem mass spectrometry (Mayo Clinic Laboratories). Samples from all time points for each subject were batch‐analysed. Detailed assay procedures are provided elsewhere [18].

### 2.5 Gene Expression

#### 2.5.1 RNA Isolation

Total RNA was isolated from vastus lateralis biopsies obtained at baseline and after 6 months using RNeasy Plus Universal Mini Kit (QIAGEN, Valencia, CA, USA) and FastPrep 24‐5G homogenizer (MP Biomedicals, Santa Ana, CA, USA) following the manufacturer's instructions. The quality and quantity of total RNA were analysed by using both, nanodrop and Bioanalyzer 2100 (Agilent Technologies, Santa Clara, CA, USA).

#### 2.5.2 Next Generation Sequencing (NGS)

One μg RNA per sample was used for the RNA sample preparations. The gold standard for RNA integrity analysis is the RNA Integrity Number (RIN) measured by Bioanalyzer. RIN values range from 1 to 10, with 1 being the poorest and 10 the highest quality [22]. To ensure high‐quality RNA sequencing, only RNAs with RINs of 9–10 were used. Sequencing libraries were generated using NEBNext® Ultra TM RNA Library Prep Kit for Illumina® (NEB, USA) following the manufacturer's recommendations, and index codes were added to attribute sequences to each sample. Briefly, mRNA was purified from total RNA, fragmented, and cDNA was then synthesized. In order to select cDNA fragments of preferentially 150–200 bp in length, the library fragments were purified with the AMPure XP system (Beckman Coulter, Beverly, USA). PCR was performed with Phusion High‐Fidelity DNA polymerase, Universal PCR primers, and Index (X) Primer. At last, PCR products were purified (AMPure XP system) and library quality was assessed on the Agilent Bioanalyzer 2100 system. To guarantee the reliability of the data, quality control (QC) was performed at each step of the procedure. Raw data (raw reads) of FASTQ format were first processed through fast. All the downstream analyses were based on clean data with high quality. Reference genome and gene model annotation files were downloaded from the genome website browser (NCBI/UCSC/Ensembl) directly. Paired‐end clean reads were aligned to the reference genome using the Spliced Transcripts Alignment to a Reference (STAR) software. STAR exhibits better alignment precision and sensitivity than other RNA‐seq aligners for both experimental and simulated data. FeatureCounts was used to count the read numbers mapped of each gene. RPKM of each gene was calculated based on the length of the gene and the read count mapped to this gene.

#### 2.5.3 Reverse Transcription Polymerase Chain Reaction (RT‐PCR)

In order to carry out the quantitative real‐time RT‐PCR, 200 ng of total RNA was reverse‐transcribed using SuperScript VILO Master Mix (Invitrogen, Carlsbad, CA, USA) according to product protocol (25°C for 10 min, 42°C for 120 min, and 85°C for 5 min), and subsequently used for TaqMan‐based real‐time PCR analysis (Applied Biosystems, Carlsbad, CA). The ratio of absorbance at 260 and 280 nm (A260/280) was used to assess the purity of isolated RNA. We only reverse‐transcribed RNA with yields ranging from 1.9 to 2, excluding from the analysis those samples not reaching this threshold. FAM labelled TaqMan Gene expression assays (Applied Biosystem, College Station, TX, USA) for WNT4 (assay ID: Hs01573505_m1), MYOD1 (assay ID: Hs00159528_m1), SCN3B (assay ID: Hs01024483_m1), DSC2 (assay ID: Hs00951428_m1), MYPBH (assay ID: Hs00192226_m1), and VIC labelled TaqMan gene expression assay for B2M (housekeeping gene, assay ID: Hs00187842_m1) and TaqMan Universal Master Mix were used following the manufacturer's protocol (50°C for 2 min and 95°C for 10 min; 40 cycles of 95°C for 15 s and 60°C for 1 min). Relative quantification (DDCT gene expression at 6 months vs. gene expression at baseline adjusted for housekeeping gene) and data analysis were performed using Real‐Time PCR system QuantStudio5 and QuantStudio Design & Analysis Software 1.3.1, respectively.

### 2.6 Statistical Analyses

Baseline characteristics were compared between groups by using independent *t*‐test or Fisher's exact test. Longitudinal changes between groups in hormones, physical function, body composition, and gene expression by RT‐PCR were tested using repeated measures ANCOVA with baseline values as covariates (SAS Software version 9.4). *p* values of < 0.05 were considered statistically significant. For differentially expressed gene analyses, paired‐sample *t*‐tests were used to compare the within‐group treatment effects (EdgR R package). Genes with adjusted *p*‐value < 0.05 and 6‐months‐to‐baseline log2fold‐change ≥ 0.5 were considered significantly differentially expressed. The *p*‐values were adjusted using Benjamini and Hochberg's approach for controlling the False Discovery Rate. For identifying regulated biologic process, Gene Ontology (GO) enrichment analyses (http://www.geneontology.org/) were used to compare the within‐group treatment effects (ClusterProfiler R package). Significance was accepted at adjusted *p*‐value < 0.05.

## 3 Results

### 3.1 Characteristics of Participants and the Effects of Interventions

Baseline characteristics with respect to age, race/ethnicity, BMI and PPT score did not differ between groups (Table 1). After 6 months, the total testosterone increased more in the LT+TRT compared with the LT+Pbo group (330.8 ± 37.4 [134% change] vs. 95.5 ± 35.5 ng/dL [38% change], *p* = 0.01). Despite similar ~10% weight loss between the LT+TRT and LT+Pbo groups (−10.9 ± 0.8 vs. −9.3 ± 0.8%, *p* = 0.30), lean body mass (−1.0 ± 0.3 [2% change] vs. −2.5 ± 0.3 kg [4% change], *p* = 0.04) and thigh muscle volume (−23.6 ± 10.9 [2% change] vs. −68.7 ± 10.3 cm^3^ [4% change], *p* = 0.04) decreased less in the LT+TRT than in the LT+Pbo group. Hip BMD was preserved in the LT+TRT group compared with the LT+Pbo group (0.005 ± 0.007 [0.4% change] vs −0.015 ± 0.004 g/cm^2^ [1.3% change], *p* = 0.03). Total 1‐RM strength increased similarly in the LT+TRT and LT+Pbo groups (71 ± 7 [23% change] vs. 74 ± 7 [24% change] kg, *p* = 0.95).

**TABLE 1:** Baseline characteristics and effect of lifestyle therapy ± TRT.

	LT+TRT (*n* = 18)	LT+Pbo (*n* = 20)	Between group *p* value*
Age (yr.)	76.7 ± 0.7	77.1 ± 0.8	0.75
Race/ethnicity, n (%)			
White	7 (39)	9 (45)	0.75
Black	9 (50)	8 (40)	0.74
Hispanic	2 (11)	3 (15)	1.00
Body mass index (kg/m^2^)	36.1 ± 1.4	35.9 ± 0.9	0.92
Physical performance test (score)	29.5 ± 1.5	28.7 ± 0.3	0.23
Total testosterone (ng/dL)			
Baseline	247.0 ± 16.0	251.1 ± 20.1	
Change at 6 months	330.8 ± 37.4^†^	95.5 ± 35.5^†^	**0.005**
Body weight (kg)			
Baseline	109.3 ± 4.8	111.9 ± 3.4	
Change at 6 months	−10.9 ± 0.8^†^	−9.3 ± 0.8^†^	0.30
Lean body mass (kg)			
Baseline	65.1 ± 1.6	67.6 ± 1.7	
Change at 6 months	−1.0 ± 0.3^†^	−2.5 ± 0.3^†^	**0.04**
Thigh muscle volume (cm^3^)			
Baseline	1591.4 ± 79.2	1658.9 ± 42.5	
Change at 6 months	−23.6 ± 10.9	−68.7 ± 10.3^†^	**0.04**
BMD at the total hip (gm/cm^2^)			
Baseline	1.126 ± 0.046	1.116 ± 0.028	
Change at 6 months	0.005 ± 0.007	−0.015 ± 0.004^†^	**0.03**
Total 1‐RM strength (kg)			
Baseline	315.8 ± 12.3	314.5 ± 16.3	
Change at 6 months	71.4 ± 7.4^†^	74.3 ± 6.8^†^	0.95

*Note:* Plus‐minus values for the change scores are the least‐squares adjusted means ± SE from the repeated‐measures analyses of variance; plus–minus values for the baseline values are the observed means ± SE. Values in bold emphasis refer to the significant *p*‐values (i.e., < 0.05).

Abbreviations: B/W, between groups; BMD, bone mineral density, LT+Pbo, lifestyle therapy (diet and exercise training) plus placebo; LT+TRT, lifestyle therapy plus TRT; 1‐RM, one‐repetition maximum.

* *p* values for the comparison between groups at baseline were calculated with use of independent *t*‐test or Fisher's exact test. *p* values for the comparison between the groups of changes from baseline to 6 months were calculated with the use of mixed‐model‐repeated‐measures analyses of variance (with baseline values as covariate). Bold values indicate statistically significant differences (*p* < 0.05).

† *p* < 0.05 for the comparison of the value at the follow‐up time with the baseline value within the group, as calculated with the use of mixed‐model repeated measures analyses of variance.

### 3.2 Differentially Expressed Genes Analyses

#### 3.2.1 The Global Muscle Transcriptome Profile Changed Differently Pre‐ and Post‐Intervention in the Two Groups

**FIGURE 2:**
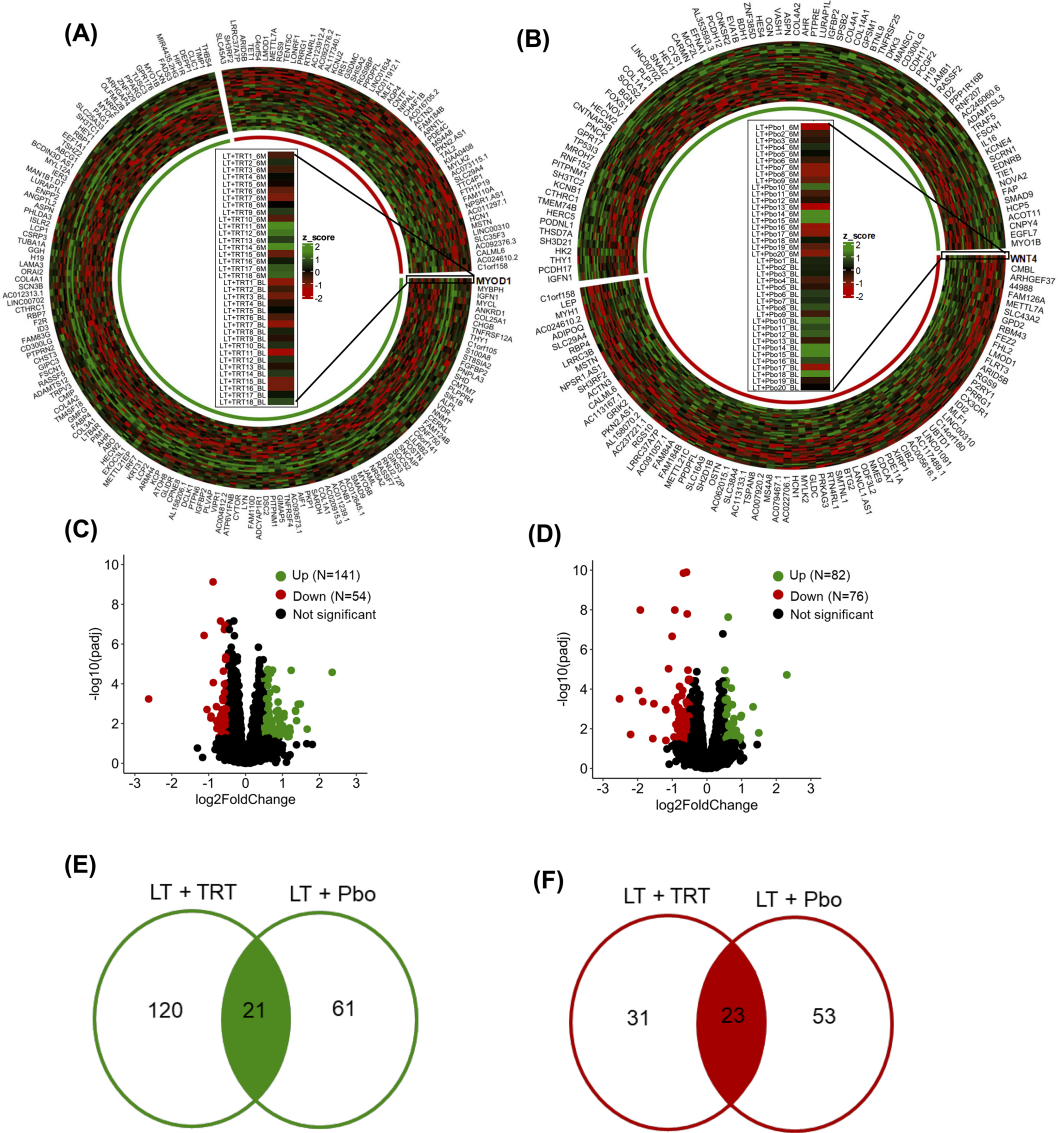
Differential gene expression analysis within groups comparing between 6 months and baseline. Heatmap of all DEGs from LT+TRT (A) and LT+Pbo (B). MYOD1 in LT+TRT and WNT4 in LT +Pbo are reported as representative genes. The numbers in the bar indicate each subject at baseline and after 6 months. Volcano plot showing DEGs in LT+TRT (C) and LT+Pbo (D). Venn diagram showing the upregulated (E) and downregulated (F) genes across both groups.

We performed the Next Generation Sequencing (RNA‐seq) to compare the global muscle transcriptomic profile within the two groups at baseline and after six months of interventions. Based on the RNA‐seq, a total of 39 160 in the LT+TRT group and 39 115 genes in the LT+Pbo group were detected. Among them, 195 were differentially expressed genes (DEGs) in the LT+TRT group (Figure 2A) and 151 in the LT+Pbo group (Figure 2B). The LT+TRT group showed a predominance of upregulated genes (Figure 2C), whereas the LT+Pbo group showed a near even split with ~50% of genes upregulated and downregulated (Figure 2D). Twenty‐one of the upregulated genes in the LT+TRT group overlapped with the upregulated genes in the LT+Pbo group (Figure 2E). Twenty‐three of the downregulated genes in the LT+Pbo group overlapped with the downregulated genes in the LT+TRT group (Figure 2F). A detailed list of the upregulated and downregulated genes within each group is provided in Table 2 and a description of each gene in Table S1.

**TABLE 2:** Upregulated and downregulated genes in the LT+TRT and LT+Pbo groups.

LT+TRT					Overlapping		LT+Pbo			
Upregulated				Downregulated	Upregulated	Downregulated	Upregulated		Downregulated	
ABCG1	ENPP2	GIMAP5	PIM1	AC011912.1	AHR	ACTN3	ACOT11	PCDH17	AC007920.2	ODF3L2
ABO	EXOC3L1	GINS3	PKP1	AC016705.2	ASPN	ARID5B	ADAMTSL3	PCGF2	AC022706.1	OSTN
AC004812.2	F2R	GIPC3	PLPPR4	AC073115.1	CD300LG	C1orf158	AL353593.3	PLP1	AC062015.1	P2RY1
AC011239.1	FABP4	GLIDR	PLVAP	AC092376.2	COL1A1	CALML6	BDH1	PNCK	AC079467.1	PDE11A
AC012313.1	FADS3	GMFG	PNPLA3	AC092376.3	COL4A1	FAM184B	BGN	PODNL1	AC091057.1	PRKAG3
AC012645.1	FAM110D	GPR176	POSTN	AC123912.4	COL4A2	HCN1	BTNL9	PPP1R16B	AC113133.1	RBM43
AC020915.3	FAM124B	HIP1R	PPARG	AL117340.1	CTHRC1	LINC00310	CARMN	PTPRE	AC113167.1	RBP4
AC093673.1	FAM83G	ID3	PTPN6	AQP4	FSCN1	LMOD1	CDH11	RASSF2	AC117489.1	RGS10
ADAMTS12	FGFBP2	IER3	PTPRN2	ARNTL	H19	LRRC37A7P	CNKSR2	RNF152	AC237221.1	SH2D1B
ADCYAP1R1	GGH	IRF7	RASSF5	C4orf54	HECW2	METTL7A	CNPY4	RNF207	ADIPOQ	SLC16A9
AIF1	GIMAP5	ISLR2	RASSF7	CHAF1B	HEY1	MLF1	CNTNAP3B	SCRN1	AL158070.2	SLC38A4
AL158206.1	GINS3	JAML	RBP1	CNTF	IGFBP2	MS4A8	COL14A1	SH3D21	ARHGEF37	SLC43A2
ALPL	GIPC3	KCP	RBP7	FAM110A	IGFN1	MSTN	CYS1	SH3TC2	BTG2	SMTNL1
ANGPTL2	CHGB	KRT31	RNU2‐72P	FTH1P19	KCNB1	MYLK2	DKK3	SNAI2	C14orf180	TSPAN8
ANKRD1	CHST3	LAMA3	S100A8	GSDMC	LINC00702	NPSR1‐AS1	EDNRB	SPSB2	CDCA7	UBTD1
ARHGAP4	CLIC1	LCP1	SARDH	IRS1	LURAP1L	PKN2‐AS1	EFNA1	THSD7A	CIB2	XIRP1
ARMH4	CMIP	LCP2	SCN3B	KCNJ2	MYO1B	PPDPFL	EGFL7	TIE1	CMBL	44 988
ATOH8	CMTM7	LILRB2	SH3TC1	KIAA0408	PITPNM1	PRRG1	EVA1B	TMEM74B	CX3CR1	
ATP6V1FNB	COL25A1	LTB4R	SHD	LINC01634	SMAD9	RGS9	FAP	TNFRSF25	FAM126A	
BCDIN3D‐AS1	COL3A1	LXN	SIK1B	LONRF1	SOCS2	RTN4RL1	FOXS1	TP53I3	FAM84A	
C1orf105	CPNE8	LYN	SLC25A33	NIPAL1	THY1	SH3RF2	GPR17	TRAF5	FEZ2	
C6orf141	CSRP3	MAN1B1‐DT	SNCAIP	PDE4C		SLC29A4	GPSM1	VASH1	FHL2	
CERKL	CYTOR	METTL21EP	ST8SIA2	RGS9BP			HCP5	ZNF385D	FLRT3	
CHGB	DCLK1	MIR4435‐2HG	THBS4	SHISA2			HERC5		GLDC	
CHST3	DEPP1	MYBPH	TIMP1	SLC35F3			HES4		GPD2	
CLIC1	DSC2	MYCL	TM4SF18	SLC45A3			HK2		GRIK2	
CMIP	EEF1A1	MYL12A	TNFRSF12A	TAL2			ID2		IDI2	
CMTM7	ENPP2	MYO5B	TNFRSF4	TENT5C			IL16		LANCL1‐AS1	
COL25A1	EXOC3L1	MYOD1	TRPV3	TET1			KCNE4		LEP	
COL3A1	F2R	MYOF	TSHZ2	TTC4P1			LAMB1		LINC01091	
CPNE8	FABP4	NNMT	TUBA1A				MANSC1		LRRC3B	
CSRP3	FADS3	NR5A2	TUSC3				MCF2L		METTL21C	
CYTOR	FAM110D	NRP2	VDR				MROH7		MYH1	
DCLK1	FAM124B	OLFML2B	VIPR1				NOV		NME9	
DEPP1	FAM83G	ORAI2	ZNF329				NOVA2		WNT4	
DSC2	FGFBP2	PAG1	ZNF750				OGN		XIRP1	
EEF1A1	GGH	PHLDA3					PCDH12		44 988	

### 3.3 Pathway Analyses

#### 3.3.1 Pathway Analyses Unveil Decreased Downregulation of Muscle and Bone‐Related Biological Pathways in LT+TRT

**FIGURE 3:**
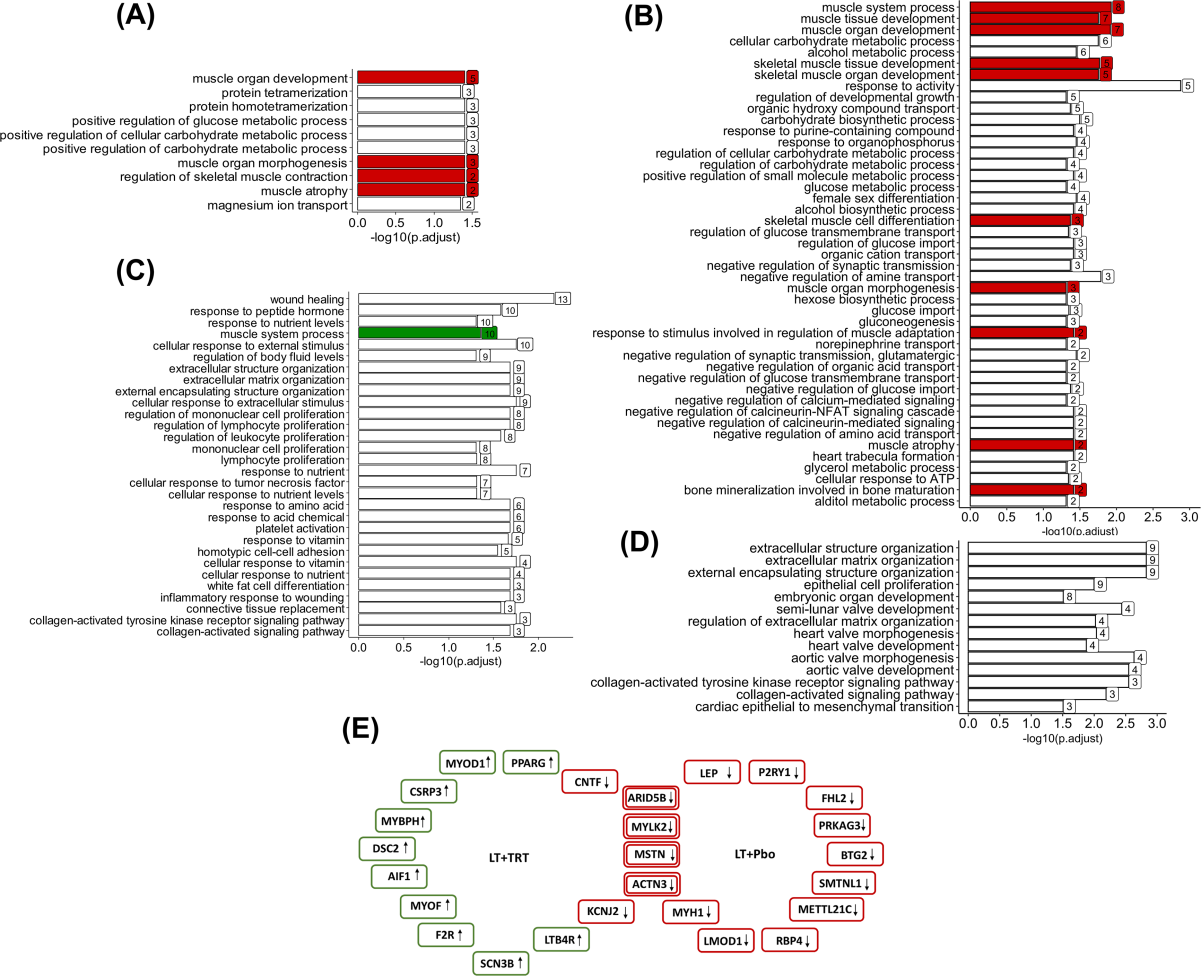
GO enrichment analysis within the two groups. Biological pathways of down‐ and upregulated DEGs in LT+TRT (A and C) and LT+Pbo (B and D). Downregulated pathways associated with muscle and bone are highlighted in red; upregulated pathway is highlighted in green. For each group (E), downregulated (green) and upregulated (red) muscle‐related genes are shown. Common genes are indicated by double outlining.

GO enrichment analysis was used to identify significantly regulated biological ontologies. All significant biological pathways are shown in Figure 3A–D, with a focus on highlighting muscle and bone pathways. GO pathway analyses revealed that in the LT+TRT group, just four muscle‐related biological pathways (muscle organ development, muscle organ morphogenesis, regulation of skeletal muscle contraction, muscle atrophy) were downregulated (Figure 3A), and one pathway (muscle system process) was upregulated (Figure 3C). In contrast, in the LT+Pbo group, nine pathways related to muscle (muscle system process, muscle tissue development, muscle organ development, skeletal muscle tissue development, skeletal muscle organ development, skeletal muscle cell differentiation, muscle organ morphogenesis, response to stimuli involved in regulation of muscle adaptation, muscle atrophy), and one pathway related to bone (bone mineralization involved in bone maturation) was downregulated (Figure 3B). None of the upregulated pathways in the LT+Pbo group were related to muscle or bone (Figure 3D). Notably, muscle system process was upregulated in the LT+TRT (Figure 3C) but downregulated in the LT+Pbo group (Figure 3B). In the LT+TRT group, 10 genes specifically involved in muscle‐related biological pathways were uniquely upregulated (Figure 3E). In contrast, in the LT+Pbo group, 10 genes involved in muscle‐related biological pathways were uniquely downregulated, with no genes upregulated (Figure 3E). Both LT+Test and LT+Pbo triggered an upregulation of extracellular organization (extracellular matrix organization, extracellular structure organization, external encapsulating structure organization) and collagen activation (collagen‐activated tyrosine kinase receptor signalling pathway, collagen‐activated signalling pathway) pathways and a downregulation of muscle development, atrophy, and organ morphogenesis (Figure 3A–D).

### 3.4 RT‐PCR Gene Expression of Muscle and Bone‐Related Genes

**FIGURE 4:**
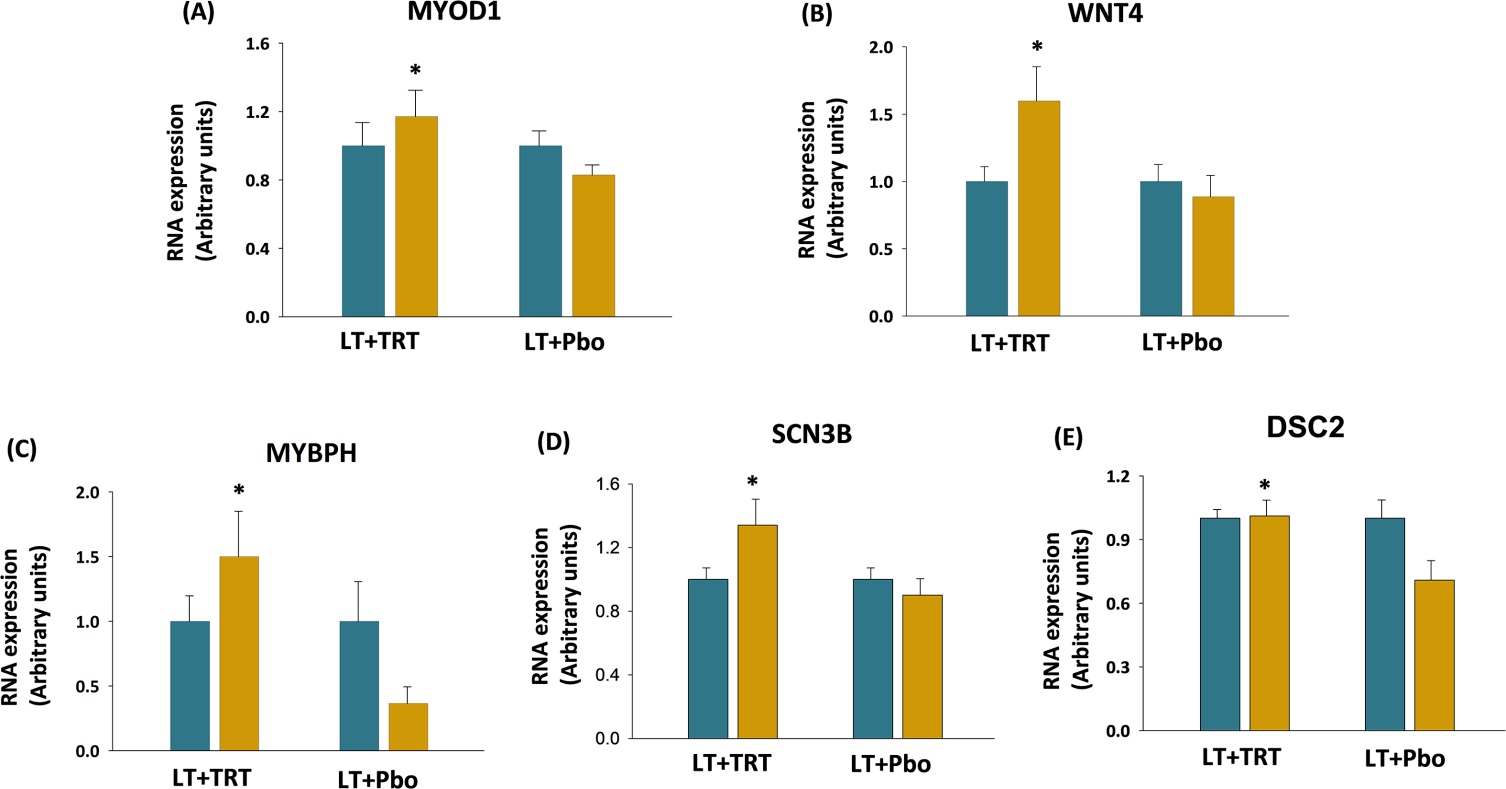
RT‐PCR of DEGs involved in muscle system process and bone metabolism. Gene expressions of MYOD1 (A), WNT4 (B), MYBPH (C), SCN3B (D), and DSC2 (E) at baseline (blue bar) and after 6 months (amber bar). Values are mean ± SE. Values are normalized (=1.0) to baseline for each participant and corrected for the expression of housekeeping gene (B2M). *Change from baseline was significantly different between groups.

Based on the results of the DEG and pathway analyses, we proceeded to investigate directly at the skeletal muscle level, the expression of the main genes involved in muscle and bone mechanisms. The RT‐PCR showed that LT+TRT resulted in a higher expression of MYOD1 (*p* = 0.02) and WNT4 (*p* = 0.02), key genes involved in muscle and bone metabolism, respectively, compared with LT+Pbo (Figure 4A,B). Furthermore, we observed significantly higher mRNA levels of MYBPH (*p* = 0.006), SCN3B (*p* = 0.02) and DSC2 (*p* = 0.01), genes involved in the muscle system process, in response to LT+TRT compared with LT+Pbo (Figure 4C–E).

## 4 Discussion

The LITROS muscle transcriptomic substudy aimed to clarify clinical outcomes with molecular prospects, providing a comprehensive insight into the intricate relationship between therapeutic interventions and musculoskeletal health in frail, older adults with obesity and hypogonadism. Our investigation is founded on the clinical basis demonstrated by the LITROS trial, showing that the combination of TRT and LT significantly contributes to attenuating the reduction of muscle and bone mass induced by weight loss [18]. Several studies have explored the impact of CR [23, 24], physical activity (PA) [25, 26, 27, 28, 29, 30], or anabolic androgenic steroids [31] on the gene expression profile. However, to the best of our knowledge, this is the first study that investigates the interplay between CR, PA, and TRT, and their collective influence on the human muscle transcriptome in this high‐risk older population.

We found distinct changes in the global muscle transcriptomic profile pre‐ and post‐intervention, depending on whether LT was used alone or combined with TRT. Specifically, our analysis has revealed that despite the LT+TRT group showing only a moderate increase in the number of DEGs compared with the LT+Pbo group, the relevant changes were linked to the functional interaction of the involved genes. TRT, combined with LT, may regulate genes involved in muscle and bone pathways, thus highlighting the intricate interaction between musculoskeletal tissues and the network of autocrine and paracrine actions [32]. In the LT+TRT group, the downregulation of muscle‐related pathways, such as muscle organ development and morphogenesis, suggests a strategic tuning of processes that regulate the formation and stabilization of new muscle. This modulation appears to prioritize the optimization and preservation of existing muscle structures over the generation of new muscle. Indeed, the upregulation of muscle system process pathway in the LT+TRT group is a phenomenon linked to multiple genes, some of which are not exclusively connected to muscle metabolism but contribute to the overall enhancement of the muscular process. Based on the muscle system process pathway, we tested the effect of the interventions on MYOD1, MYBPH, SCN3B, and DSC2 gene expression using RT‐PCR to confirm our results. Notably, we showed a higher expression of MYOD1 in response to LT+TRT compared with LT+Pbo group. MYOD1 is a key regulator of skeletal myogenesis that directs contractile protein synthesis. Specifically, it has been shown that MYOD1 is a regulator of the metabolic capacity of mature skeletal muscle to ensure that sufficient energy is available to support muscle contraction [33]. In vitro, Wang et al. showed that inducing MyoD expression in brown preadipocytes inhibits brown adipogenesis and upregulates the expression of myogenic genes. This finding designates MyoD as a critical negative regulator of brown adipocyte development, thereby favouring differentiation towards myoblasts [34]. At the clinical level, it is noteworthy that, albeit to a moderate extent, the LT+TRT group continues to manifest a decline in muscle mass, possibly accounted by the downregulation of genes associated with muscle anabolism and the insufficiency of counterregulatory mechanisms.

We observed a downregulation of genes involved in regulation of skeletal muscle ction pathway in LT+TRT group. This finding is consistent with that of Haren MT et al., who also reported a downregulation of contraction‐related genes in response to testosterone in mouse skeletal muscle [35]. They demonstrated that testosterone modulates gene expression pathways involved in muscle contraction, nutrient accumulation, and protein turnover, highlighting a complex response to hormonal treatment. Testosterone‐induced gene expression changes may optimize muscle function and recovery while managing stress‐induced damage. The reduction in contractile activity may be a strategy to minimize stress and workload on skeletal muscle, thereby preserving existing muscle tissue and avoiding the acceleration of atrophy. Recently, Russell et al. showed that even modest reduction in levels of muscle contraction (< 15%) was sufficient to protect dystrophic skeletal muscles in murine models of Duchenne muscular dystrophy, showcasing a potential avenue to attenuate stress‐induced damage [36].

On the other hand, the higher number of genes involved in commonly downregulated muscle‐related pathways highlighted a more pronounced downregulation in the LT+Pbo group. This phenomenon, reflected in the downregulation of several muscle‐related pathways such as skeletal muscle tissue, muscle tissue, and skeletal muscle organ development, is not counterbalanced by positive regulatory pathways, as observed in LT+TRT. This implies a diminished capacity of the muscle to coordinate its functions at a broader physiological level. Moreover, the LT+Pbo group showed downregulation of response to stimulus involved in the regulation of muscle adaptation that can be explained by the downregulation of skeletal muscle cell differentiation. This process involves the transition of skeletal muscle cells from an undifferentiated to a specialized state, characteristic of mature muscle cells [37]. The transition of muscle cells assumes a pivotal role in the regeneration, and repair of muscle tissue [38]. In vitro studies have recently demonstrated that dihydrotestosterone and anabolic steroids promote the proliferation and differentiation of the mouse skeletal muscle myoblast C2C12 cell line [39]. In this context, testosterone, as evidenced by its absence in the downregulation of this pathway in the LT+TRT group, may exert a modulatory influence on this crucial differentiation process, thereby contributing to the preservation of muscle tissue's structural and functional integrity [40, S1].

Recently, it has been demonstrated that during exercise, the α2/β2/γ3 heterotrimer of the energy‐sensing AMP‐activated protein kinase (AMPK) protein complex is predominantly activated and can monitor cellular energy status and mitochondrial biogenesis [S2]. In addition, it has been shown that during CR, AMPK is upregulated in human skeletal muscle [24]. The activity of the AMPK complex is primarily associated with the phosphorylation of the skeletal muscle‐specific subunit, AMP‐activated protein kinase non‐catalytic subunit gamma 3 (PRKAG3) [S2]. Recent findings indicate a downregulation in the muscle gene expression of PRKAG3 in older individuals compared to younger individuals with similar PA levels. This suggests that the activation of the AMPK complex in response to exercise may be diminished in aging muscles [S3] while such activation may be restored during CR [24]. In our study, we observed no significant differences in the gene expression of PRKAG3 in the LT+TRT group pre‐ and post‐intervention, while a downregulation was evident in the LT+Pbo group. This implies that the inclusion of TRT during LT may play a role in mitigating the age‐induced decline in activation of the AMPK complex during exercise in older individuals. In addition, Mohamed et al. showed that in aged mice, exercise over activates PARP‐1 thereby inhibiting SIRT‐1 activity, [S4], a key gene that regulates some of the CR‐responsive biological pathways [S5] that results in reduced mitochondrial biogenesis and metabolism [S4]. In contrast with Mohamed et al., we did not show any changes in the gene expression of SIRT1, either when LT was administered alone or in conjunction with TRT.

Interestingly, in line with the clinical evidence shown in the LITROS trial, we also observed the downregulation of bone mineralization involved in bone maturation pathway in the LT+Pbo group. The decreased mineralization could result in less dense bone tissue, making it more susceptible to fragility and fractures [S6]. The absence of specific downregulated bone‐related pathways in the LT+TRT group suggests that testosterone may play a crucial role in maintaining BMD by regulating the bone mineralization process, confirming the importance of testosterone's paracrine action in improving bone quality [7]. In the context of bone metabolism, it is well known that Wnt β‐catenin‐dependent signalling is the main pathway involved in bone metabolism and its activation, through the binding of WNTs proteins to its co‐receptors, LRP5/6, lead to bone formation [S7, S8]. However, Wnt signalling also includes β‐catenin‐independent signalling that regulates a multitude of cellular processes [S9]. We found a higher gene expression of WNT4 in response to LT+TRT group compared with LT+Pbo. In the context of our investigation, the current state of knowledge regarding WNT4 remains limited. WNT4 plays a crucial role in promoting the osteogenic differentiation of bone marrow stromal cells and can activate both Wnt β‐catenin‐dependent and β‐catenin‐independent signalling based on different cellular processes [S10]. Specifically, Gozo et al. demonstrated that in myoblast cells, FOCX2 triggered WNT signalling by directly interacting with the WNT4 promoter region. This led to increased expression of bone morphogenetic protein‐4 (BMP4), which in turn suppressed myogenesis, consequently influencing their lineage commitment towards osteogenesis [S11]. Recently, Bo Yu et al., showed that WNT4 enhances bone formation and inhibits osteoclast formation and inflammation in osteoporosis and skeletal aging mouse models by inhibiting nuclear factor‐kB (NF‐kB) via β‐catenin‐independent signalling [S12]. To our knowledge, we demonstrate for the first time that TRT upregulates WNT4 to exert a paracrine effect thereby preserving BMD during LT. These findings highlight the close interaction between muscle and bone, and the influence of TRT on both tissues. Moreover, it is well known that collagen is the most abundant protein in bone tissue [S13]. In this regard, we showed an overlap between the two groups in the upregulated gene expression of COL1A1, the gene encoding for Type I Collagen, in addition to COL4A2 and COL4A1, which belong to the same family of genes. Interestingly, those genes have been identified by Miyamoto‐Mikami et al., as common genes observed in both High‐Intensity Interval Training (HIIT) and resistance training [25]. Indeed, Robinson et al. reported that 30% of HIIT‐induced genes are shared with resistance training.

Our investigation exhibits commendable strengths and acknowledges certain limitations that shape its overall scientific merit. Our study's strength lies in its randomized, double‐blind design, providing reliable results and minimizing potential biases. The inclusion of a distinctive patient cohort further enriches our findings, allowing for a nuanced understanding of specific subpopulations for broader applicability. We examined a cohort of older adults facing a cluster of challenges, including obesity, frailty, and hypogonadism, reflecting a population at high risk for musculoskeletal health decline [S15]. This real‐world representation underscores the study's potential and its direct applicability to an increasingly widespread and diverse demographic group of older adults [S16].

A key strength lies in the implementation of a rigorous LT program, closely supervised and consistent, alongside TRT. This combined approach has not only contributed to a relatively low dropout rate but also underscores the relevance of these interventions to real‐world scenarios. The meticulous nature of our LT program, coupled with the advanced molecular insights provided by NGS, particularly RNA sequencing, adds depth to our analysis, enhancing the comprehensiveness of our research. The LITROS muscle transcriptomic substudy took advantage of state‐of‐the‐art NGS to assess changes in the global muscle transcriptomic profile [S17]. This advanced technique facilitated the identification of DEGs and their related pathways directly on skeletal muscle tissue, allowing us to gain insights into the molecular underpinnings of the observed clinical effects.

However, our study also has limitations. The modest size of our sample poses a challenge to the generalizability of our findings and the strength of our conclusions. The recruitment for this study has been completed, making it impossible to include more data or expand the sample size. Additionally, the relatively brief follow‐up period restricts our ability to assess the long‐term efficacy of the adopted therapies and the sustainability of observed results. Ethical considerations led to the omission of a testosterone‐only treatment group, introducing a challenge in untangling the specific contributions of TRT versus LT to the observed muscle transcriptome outcomes Despite these limitations, we believe it was necessary to provide the benefits of LT to both study groups. We employed a multicomponent intervention consisting of diet, exercise, and testosterone—the opposing effects of diet vs. exercise and testosterone on muscle‐ and bone‐related genes may have contributed to attenuation of changes in gene expression in response to the intervention. Our study did not include fibre typing to separately analyse the effects of resistance versus aerobic exercise within the intervention. Resistance training typically increases Type II (fast‐twitch) fibres [S18], while aerobic exercise tends to enhance Type I (slow twitch) fibres [S19]. Although we did not directly measure muscle fibre types, our analyses controlled various factors related to muscle health and physical performance. We believe the combined exercise regimen, which was an integral part of the lifestyle intervention, likely provided a balanced effect on muscle fibre types, which could have helped mitigate some variability. A significant challenge highlighted in our study is the difficulty in maintaining adherence to both interventions, as evidenced by the 60%–70% discontinuation rate of any form of TRT within a year from the initial prescription [S60]. This mirrors the real‐world scenario where sustaining long‐term adherence proves to be a complex task.

## 5 Conclusion

Our research represents a union of clinical relevance, therapeutic approaches, and advanced molecular techniques, as an integration of clinical and molecular aspects. Our study supports the conclusion that the addition of TRT to LT effectively mitigates the weight loss‐induced reduction of muscle mass and BMD by countering the weight loss‐induced downregulation of genes involved in muscle and bone anabolism. Future inquiries in this domain could build upon our study, further illuminating the path towards healthier aging and improved musculoskeletal outcomes. Our findings underscore the potential for more targeted and effective strategies in addressing the complex health challenges of older adults with obesity and hypogonadism.

## Ethics Statement

All authors certify that the manuscript complies with the ethical guidelines for authorship and publishing in the Journal of Cachexia, Sarcopenia and Muscle. All human studies have been approved by the appropriate ethics committee and have therefore been performed in accordance with the ethical standards laid down in the 1964 Declaration of Helsinki and its later amendments. All persons gave their informed consent prior to their inclusion in the study. Details that might disclose the identity of the subjects have been omitted.

## Conflicts of Interest

The authors declare no conflicts of interest.

## Supporting information


**Table S1** Description of upregulated and downregulated genes in the LT+TRT and LT+Pbo Groups (pages 3–9).

**Data S1** Supplementary Information.
