# Supplementary material for: Testosterone Modulation of Muscle Transcriptomic Profile During Lifestyle Therapy in Older Men with Obesity and Hypogonadism
Source: J Cachexia Sarcopenia Muscle. 2025 Jan 27;16(1):e13697. doi: 10.1002/jcsm.13697 (PMC11924803; doi:10.1002/jcsm.13697)
Supplement: Supplementary file 1 — Table S1 Description of upregulated and downregulated genes in the LT+TRT and LT+Pbo Groups (pages 3–9). [file JCSM-16-e13697-s002.docx]

**Testosterone Modulation of Muscle Transcriptomic Profile during Lifestyle Therapy in Older Men with Obesity and Hypogonadism**

Viola Viola, Tagari Samanta, Maria Liza Duremdes Nava et al

**ONLINE SUPPLEMENTARY MATERIAL**

**Supplementary Table 1.** Description of Upregulated and Downregulated Genes in the LT+TRT and LT+Pbo Groups (pages 3-9)

**Supplementary References** (pages 10-11)

**Supplementary Table 1.** Description of Upregulated and Downregulated Genes in the LT+TRT and LT+Pbo Groups

**LT + TRT Upregulated Genes**

| **GENE SYMBOL** | **GENE DESCRIPTION** |
| --- | --- |
| ABCG1 | ATP-Binding Cassette, Sub-Family G (WHITE), Member 1 |
| ABO | ABO Blood Group (Transferase A, Alpha 1-3-N-Acetylgalactosaminyltransferase; Transferase B, Alpha 1-3-Galactosyltransferase) |
| AC004812.2 | RAB35 Antisense RNA 1 |
| AC011239.1 | New Transcript |
| AC012313.1 | New Transcript |
| AC012645.1 | New Transcript |
| AC020915.3 | New Transcript |
| AC093673.1 | New Transcript |
| ADAMTS12 | A Disintegrin-Like And Metalloprotease (Reprolysin Type) With Thrombospondin Type 1 Motif, 12 |
| ADCYAP1R1 | Adenylate Cyclase Activating Polypeptide 1 (Pituitary) Receptor Type I |
| AIF1 | Interferon Gamma Responsive Transcript |
| AL158206.1 | New Transcript |
| ALPL | Alkaline Phosphatase, Liver/Bone/Kidney |
| ANGPTL2 | Angiopoietin-Like Protein 2 |
| ANKRD1 | Ankyrin Repeat Domain-Containing Protein 1 |
| ARHGAP4 | Rho GTPase-Activating Protein 4 |
| ARMH4 | Armadillo-Like Helical Domain-Containing Protein 4 |
| ATOH8 | Atonal Homolog BHLH Transcription Factor 8 |
| ATP6V1FNB | Sperm Microtubule Inner Protein 1 |
| BCDIN3D-AS1 | BCDIN3D Antisense RNA 1 (Non-Protein Coding) |
| C1orf105 | Chromosome 1 Open Reading Frame 105 |
| C6orf141 | Chromosome 6 Open Reading Frame 141 |
| CERKL | Ceramide Kinase-Like Protein |
| CHGB | Chromogranin B |
| CHST3 | Carbohydrate (Chondroitin 6) Sulfotransferase 3 |
| CLIC1 | Regulatory Nuclear Chloride Ion Channel Protein |
| CMIP | C-Maf-Inducing Protein |
| CMTM7 | Chemokine-Like Factor Superfamily Member 7 |
| COL25A1 | Collagen Type XXV Alpha 1 Chain |
| COL3A1 | Collagen Type III Alpha 1 Chain |
| CPNE8 | Copine VIII |
| CSRP3 | Cysteine And Glycine-Rich Protein 3 (Cardiac LIM Protein) |
| CYTOR | Long Intergenic Non-Protein Coding RNA 152 |
| DCLK1 | Doublecortin-Like Kinase 1 |
| DEPP1 | Decidual Protein Induced By Progesterone |
| DSC2 | Desmocollin 2 |
| EEF1A1 | Eukaryotic Translation Elongation Factor 1 Alpha 1 |
| ENPP2 | Ectonucleotide Pyrophosphatase/Phosphodiesterase 2 |
| EXOC3L1 | Exocyst Complex Component 3 Like 1 |
| F2R | Coagulation Factor II Thrombin Receptor |
| FABP4 | Fatty Acid Binding Protein 4 |
| FADS3 | Fatty Acid Desaturase 3 |
| FAM110D | Family With Sequence Similarity 110 Member D |
| FAM124B | Family With Sequence Similarity 124 Member B |
| FAM83G | Family With Sequence Similarity 83 Member G |
| FGFBP2 | Fibroblast Growth Factor Binding Protein 2 |
| GGH | Gamma-Glutamyl Hydrolase |
| GIMAP5 | GTPase, IMAP Family Member 5 |
| GINS3 | DNA Replication Complex GINS Protein PSF3 |
| GIPC3 | PDZ Domain-Containing Protein GIPC3 |
| GLIDR | Glioblastoma Down-Regulated RNA |
| GMFG | Glia Maturation Factor Gamma |
| GPR176 | G Protein-Coupled Receptor 176 |
| HIP1R | Huntingtin Interacting Protein 1 Related |
| ID3 | Inhibitor Of DNA Binding 3 |
| IER3 | Immediate Early Response 3 |
| IRF7 | Interferon Regulatory Factor 7 |
| ISLR2 | Immunoglobulin Superfamily Containing Leucine Rich Repeat 2 |
| JAML | Junction Adhesion Molecule Like |
| KCP | Kielin Cysteine Rich BMP Regulator |
| KRT31 | Keratin 31 |
| LAMA3 | Laminin Subunit Alpha 3 |
| LCP1 | Lymphocyte Cytosolic Protein 1 |
| LCP2 | Lymphocyte Cytosolic Protein 2 |
| LILRB2 | Leukocyte Immunoglobulin Like Receptor B2 |
| LTB4R | Leukotriene B4 Receptor |
| LXN | Endogenous Carboxypeptidase Inhibitor |
| LYN | LYN Proto-Oncogene, Src Family Tyrosine Kinase |
| MAN1B1-DT | MAN1B1 Divergent Transcript |
| METTL21EP | Methyltransferase Like 21E, Pseudogene |
| MIR4435-2HG | MIR4435-1 Host Gene (Non-Protein Coding) |
| MYBPH | Myosin-Binding Protein H |
| MYCL | MYCL Proto-Oncogene, BHLH Transcription Factor |
| MYL12A | Myosin Regulatory Light Chain 2, Nonsarcomeric |
| MYO5B | Myosin VB |
| MYOD1 | Myogenic Differentiation 1 |
| MYOF | Myoferlin |
| NNMT | Nicotinamide N-Methyltransferase |
| NR5A2 | Nuclear Receptor Subfamily 5 Group A Member 2 |
| NRP2 | Neuropilin 2 |
| OLFML2B | Olfactomedin Like 2B |
| ORAI2 | ORAI Calcium Release-Activated Calcium Modulator 2 |
| PAG1 | Phosphoprotein Membrane Anchor With Glycosphingolipid Microdomains 1 |
| PHLDA3 | Pleckstrin Homology Like Domain Family A Member 3 |
| PIM1 | Pim-1 Proto-Oncogene, Serine/Threonine Kinase |
| PKP1 | Plakophilin 1 |
| PLPPR4 | Phospholipid Phosphatase Related 4 |
| PLVAP | Plasmalemma Vesicle Associated Protein |
| PNPLA3 | Patatin Like Phospholipase Domain Containing 3 |
| POSTN | Periostin, Osteoblast Specific Factor |
| PPARG | Peroxisome Proliferator Activated Receptor Gamma |
| PTPN6 | Protein Tyrosine Phosphatase Non-Receptor Type 6 |
| PTPRN2 | Protein Tyrosine Phosphatase Receptor Type N2 |
| RASSF5 | Ras Association Domain Family Member 5 |
| RASSF7 | Ras Association Domain Family Member 7 |
| RBP1 | Retinol Binding Protein 1 |
| RBP7 | Retinol Binding Protein 7 |
| RNU2-72P | RNA, U2 Small Nuclear 72, Pseudogene |
| S100A8 | S100 Calcium Binding Protein A8 |
| SARDH | Sarcosine Dehydrogenase |
| SCN3B | Sodium Voltage-Gated Channel Beta Subunit 3 |
| SH3TC1 | SH3 Domain And Tetratricopeptide Repeats 1 |
| SHD | Src Homology 2 Domain Containing Transforming Protein D |
| SIK1B | Salt-Inducible Kinase 1B |
| SLC25A33 | Solute Carrier Family 25 Member 33 |
| SNCAIP | Synuclein Alpha Interacting Protein |
| ST8SIA2 | ST8 Alpha-N-Acetyl-Neuraminide Alpha-2,8-Sialyltransferase 2 |
| THBS4 | Thrombospondin 4 |
| TIMP1 | Tissue Inhibitor Of Metalloproteinases 1 |
| TM4SF18 | Transmembrane 4 L Six Family Member 18 |
| TNFRSF12A | Tumor Necrosis Factor Receptor Superfamily Member 12A |
| TNFRSF4 | Tumor Necrosis Factor Receptor Superfamily Member 4 |
| TRPV3 | Transient Receptor Potential Cation Channel, Subfamily V, Member 3 |
| TSHZ2 | Teashirt Zinc Finger Homeobox 2 |
| TUBA1A | Tubulin Alpha 1a |
| TUSC3 | Tumor Suppressor Candidate 3 |
| VDR | Vitamin D Receptor |
| VIPR1 | Vasoactive Intestinal Peptide Receptor 1 |
| ZNF329 | Zinc Finger Protein 329 |
| ZNF750 | Zinc Finger Protein 750 |

**LT+PBO Upregulated Genes**

| **GENE SYMBOL** | **GENE DESCRIPTION** |
| --- | --- |
| AC245060.6 | New Transcript |
| ACOT11 | Acyl-CoA Thioesterase 11 |
| ADAMTSL3 | A Disintegrin-Like And Metalloprotease Domain With Thrombospondin Type I Motifs-Like 3 |
| AL353593.3 | New Transcript |
| BDH1 | 3-Hydroxybutyrate Dehydrogenase 1 |
| BGN | Biglycan |
| BTNL9 | Butyrophilin Like 9 |
| CARMN | Cardiac Mesoderm Enhancer-Associated Non-Coding RNA |
| CDH11 | Cadherin 11 |
| CNKSR2 | Connector Enhancer Of Kinase Suppressor Of Ras 2 |
| CNPY4 | Canopy FGF Signaling Regulator 4 |
| CNTNAP3B | Contactin Associated Protein Family Member 3B |
| COL14A1 | Collagen Type XIV Alpha 1 Chain |
| CYS1 | Cystin 1 |
| DKK3 | Dickkopf WNT Signaling Pathway Inhibitor 3 |
| EDNRB | Endothelin Receptor Type B |
| EFNA1 | EPH-Related Receptor Tyrosine Kinase Ligand 1 |
| EGFL7 | Multiple Epidermal Growth Factor-Like Domains Protein 7 |
| EVA1B | Eva-1 Homolog B |
| FAP | Fibroblast Activation Protein Alpha |
| FOXS1 | Forkhead Box S1 |
| GPR17 | G Protein-Coupled Receptor 17 |
| GPSM1 | G Protein Signaling Modulator 1 |
| HCP5 | HLA Class I Histocompatibility Antigen Protein P5 |
| HERC5 | HECT And RLD Domain Containing E3 Ubiquitin Protein Ligase 5 |
| HES4 | Hes Family BHLH Transcription Factor 4 |
| HK2 | Muscle Form Hexokinase |
| ID2 | Inhibitor Of DNA Binding 2 |
| IL16 | Interleukin 16 |
| KCNE4 | Potassium Voltage-Gated Channel Subfamily E Regulatory Subunit 4 |
| LAMB1 | Laminin Subunit Beta 1 |
| MANSC1 | MANSC Domain Containing 1 |
| MCF2L | MCF.2 Cell Line Derived Transforming Sequence Like |
| MROH7 | Maestro Heat-Like Repeat-Containing Protein Family Member 7 |
| NOV | Nephroblastoma-Overexpressed Gene Protein Homolog |
| NOVA2 | Neuro-Oncological Ventral Antigen 2 |
| OGN | Osteoglycin |
| PCDH12 | Protocadherin 12 |
| PCDH17 | Protocadherin 17 |
| PCGF2 | Polycomb Group Ring Finger 2 |
| PLP1 | Proteolipid Protein 1 |
| PNCK | Pregnancy Up-Regulated Nonubiquitous CaM Kinase |
| PODNL1 | Podocan Like 1 |
| PPP1R16B | Protein Phosphatase 1 Regulatory Subunit 16B |
| PTPRE | Protein Tyrosine Phosphatase Receptor Type E |
| RASSF2 | Ras Association Domain Family Member 2 |
| RNF152 | Ring Finger Protein 152 |
| RNF207 | Ring Finger Protein 207 |
| SCRN1 | Secernin 1 |
| SH3D21 | SH3 Domain Containing 21 |
| SH3TC2 | SH3 Domain And Tetratricopeptide Repeats 2 |
| SNAI2 | Snail Family Transcriptional Repressor 2 |
| SPSB2 | SplA/Ryanodine Receptor Domain And SOCS Box Containing 2 |
| THSD7A | Thrombospondin Type 1 Domain Containing 7A |
| TIE1 | Tyrosine Kinase With Immunoglobulin Like And EGF Like Domains 1 |
| TMEM74B | Transmembrane Protein 74B |
| TNFRSF25 | Tumor Necrosis Factor Receptor Superfamily, Member 25 |
| TP53I3 | Tumor Protein P53 Inducible Protein 3 |
| TRAF5 | TNF Receptor-Associated Factor 5 |
| VASH1 | Vasohibin 1 |
| ZNF385D | Zinc Finger Protein 385D |

**Overlapped Upregulated Genes**

| **GENE SYMBOL** | **GENE DESCRIPTION** |
| --- | --- |
| AHR | Aryl Hydrocarbon Receptor |
| ASPN | Asporin |
| CD300LG | CD300 Molecule Like Family Member G |
| COL1A1 | Collagen Type I Alpha 1 Chain |
| COL4A1 | Collagen Type IV Alpha 1 Chain |
| COL4A2 | Collagen Type IV Alpha 2 Chain |
| CTHRC1 | Collagen Triple Helix Repeat Containing 1 |
| FSCN1 | Fascin Actin-Bundling Protein 1 |
| H19 | H19 Imprinted Maternally Expressed Transcript |
| HECW2 | HECT, C2 And WW Domain Containing E3 Ubiquitin Protein Ligase 2 |
| HEY1 | Hes Related Family BHLH Transcription Factor With YRPW Motif 1 |
| IGFBP2 | Insulin Like Growth Factor Binding Protein 2 |
| IGFN1 | Immunoglobulin Like And Fibronectin Type III Domain Containing 1 |
| KCNB1 | Potassium Voltage-Gated Channel Subfamily B Member 1 |
| LINC00702 | Long Intergenic Non-Protein Coding RNA 702 |
| LURAP1L | Leucine Rich Adaptor Protein 1 Like |
| MYO1B | Myosin IB |
| PITPNM1 | Phosphatidylinositol Transfer Protein Membrane Associated 1 |
| SMAD9 | SMAD Family Member 9 |
| SOCS2 | Suppressor Of Cytokine Signaling 2 |
| THY1 | Thy-1 Cell Surface Antigen |

**LT+TRT Downregulated Genes**

| **GENE SYMBOL** | **GENE DESCRIPTION** |
| --- | --- |
| AC011297.1 | New Transcript |
| AC011912.1 | New Transcript |
| AC016705.2 | New Transcript |
| AC073115.1 | New Transcript |
| AC092376.2 | New Transcript |
| AC092376.3 | New Transcript |
| AC123912.4 | New Transcript |
| AL117340.1 | New Transcript |
| AQP4 | Aquaporin 4 |
| ARNTL | Basic Helix-Loop-Helix ARNT Like 1 |
| C4orf54 | Chromosome 4 Open Reading Frame 54 |
| CHAF1B | Chromatin Assembly Factor 1 Subunit B |
| CNTF | Ciliary Neurotrophic Factor |
| FAM110A | Family With Sequence Similarity 110 Member A |
| FTH1P19 | Ferritin Heavy Chain 1 Pseudogene 19 |
| GSDMC | Gasdermin C |
| IRS1 | Insulin Receptor Substrate 1 |
| KCNJ2 | Potassium Inwardly Rectifying Channel Subfamily J Member 2 |
| KIAA0408 | Uncharacterized Protein KIAA0408 |
| LINC01634 | Long Intergenic Non-Protein Coding RNA 1634 |
| LONRF1 | LON Peptidase N-Terminal Domain And Ring Finger 1 |
| NIPAL1 | NIPA Like Domain Containing 1 |
| PDE4C | Phosphodiesterase 4C |
| RGS9BP | Regulator Of G Protein Signaling 9 Binding Protein |
| SHISA2 | Shisa Family Member 2 |
| SLC35F3 | Solute Carrier Family 35 Member F3 |
| SLC45A3 | Solute Carrier Family 45 Member 3 |
| TAL2 | TAL BHLH Transcription Factor 2 |
| TENT5C | Terminal Nucleotidyltransferase 5C |
| TET1 | Tet Methylcytosine Dioxygenase 1 |
| TTC4P1 | Tetratricopeptide Repeat Domain 4 Pseudogene 1 |

**LT+PBO Downregulated Genes**

| **GENE SYMBOL** | **GENE DESCRIPTION** |
| --- | --- |
| AC005616.1 | New Transcript |
| AC007920.2 | New Transcript |
| AC022706.1 | New Transcript |
| AC062015.1 | New Transcript |
| AC079467.1 | New Transcript |
| AC091057.1 | New Transcript |
| AC113133.1 | New Transcript |
| AC113167.1 | New Transcript |
| AC117489.1 | New Transcript |
| AC237221.1 | New Transcript |
| ADIPOQ | Adiponectin, C1Q And Collagen Domain Containing |
| AL158070.2 | New Transcript |
| ARHGEF37 | Rho Guanine Nucleotide Exchange Factor 37 |
| BTG2 | BTG Anti-Proliferation Factor 2 |
| C14orf180 | Chromosome 14 Open Reading Frame 180 |
| CDCA7 | Cell Division Cycle Associated 7 |
| CIB2 | Calcium And Integrin Binding Family Member 2 |
| CMBL | Carboxymethylenebutenolidase Homolog |
| CX3CR1 | C-X3-C Motif Chemokine Receptor 1 |
| FAM126A | Family With Sequence Similarity 126 Member A |
| FAM84A | Family With Sequence Similarity 84 Member A |
| FEZ2 | Fasciculation And Elongation Protein Zeta 2 |
| FHL2 | Four And A Half LIM Domains 2 |
| FLRT3 | Fibronectin Leucine Rich Transmembrane Protein 3 |
| GLDC | Glycine Dehydrogenase (Decarboxylating), Mitochondrial |
| GPD2 | Glycerol-3-Phosphate Dehydrogenase 2 |
| GRIK2 | Glutamate Ionotropic Receptor Kainate Type Subunit 2 |
| IDI2 | Isopentenyl-Diphosphate Delta Isomerase 2 |
| LANCL1-AS1 | LANCL1 Antisense RNA 1 |
| LEP | Leptin |
| LINC01091 | Long Intergenic Non-Protein Coding RNA 1091 |
| LRRC3B | Leucine Rich Repeat Containing 3B |
| METTL21C | Methyltransferase 21C, AARS1 Lysine |
| MYH1 | Myosin Heavy Chain 1 |
| NME9 | NME/NM23 Family Member 9 |
| ODF3L2 | Outer Dense Fiber Of Sperm Tails 3 Like 2 |
| OSTN | Osteocrin |
| P2RY1 | Purinergic Receptor P2Y1 |
| PDE11A | Phosphodiesterase 11A |
| PRKAG3 | Protein Kinase AMP-Activated Non-Catalytic Subunit Gamma 3 |
| RBM43 | RNA Binding Motif Protein 43 |
| RBP4 | Retinol Binding Protein 4 |
| RGS10 | Regulator Of G Protein Signaling 10 |
| SH2D1B | SH2 Domain Containing 1B |
| SLC16A9 | Solute Carrier Family 16 Member 9 |
| SLC38A4 | Solute Carrier Family 38 Member 4 |
| SLC43A2 | Solute Carrier Family 43 Member 2 |
| SMTNL1 | Smoothelin Like 1 |
| TSPAN8 | Tetraspanin 8 |
| UBTD1 | Ubiquitin Domain Containing 1 |
| WNT4 | Wnt Family Member 4 |
| XIRP1 | Xin Actin Binding Repeat Containing 1 |
| 44988 | Centrosomal Protein 164kDa Pseudogene 1 |

**Overlapped Downregulated Genes**

| **GENE SYMBOL** | **GENE DESCRIPTION** |
| --- | --- |
| AC024610.2 | New Transcript |
| ACTN3 | Actinin Alpha 3 |
| ARID5B | AT-Rich Interaction Domain 5B |
| C1orf158 | Uncharacterized Protein |
| CALML6 | Calmodulin Like 6 |
| FAM184B | Family With Sequence Similarity 184 Member B |
| HCN1 | Hyperpolarization Activated Cyclic Nucleotide Gated Potassium Channel 1 |
| LINC00310 | Long Intergenic Non-Protein Coding RNA 310 |
| LMOD1 | Leiomodin 1 |
| LRRC37A7P | Leucine Rich Repeat Containing 37 Member A7, Pseudogene |
| METTL7A | Methyltransferase Like 7A |
| MLF1 | Myeloid Leukemia Factor 1 |
| MS4A8 | Membrane Spanning 4-Domains A8 |
| MSTN | Myostatin |
| MYLK2 | Myosin Light Chain Kinase 2 |
| NPSR1-AS1 | NPSR1 Antisense RNA 1 |
| PKN2-AS1 | PKN2 Antisense RNA 1 |
| PPDPFL | Pancreatic Progenitor Cell Differentiation And Proliferation Factor Like |
| PRRG1 | Proline Rich And Gla Domain 1 |
| RGS9 | Regulator Of G Protein Signaling 9 |
| RTN4RL1 | Reticulon 4 Receptor Like 1 |
| SH3RF2 | SH3 Domain Containing Ring Finger 2 |
| SLC29A4 | Solute Carrier Family 29 Member 4 |

**Supplementary References**

S1. Fu R, Liu J, Fan J, Li R, Li D, Yin J, et al. Novel evidence that testosterone promotes cell proliferation and differentiation via G protein-coupled receptors in the rat L6 skeletal muscle myoblast cell line. J Cell Physiol. 2012;227:98-107.

S2. Birk JB, Wojtaszewski JF. Predominant alpha2/beta2/gamma3 AMPK activation during exercise in human skeletal muscle. J Physiol. 2006;577:1021-32.

S3. Lagerwaard B, Nieuwenhuizen AG, Bunschoten A, de Boer VCJ, Keijer J. Matrisome, innervation and oxidative metabolism affected in older compared with younger males with similar physical activity. J Cachexia Sarcopenia Muscle. 2021;12:1214-31.

S4. Mohamed JS, Wilson JC, Myers MJ, Sisson KJ, Alway SE. Dysregulation of SIRT-1 in aging mice increases skeletal muscle fatigue by a PARP-1-dependent mechanism. Aging (Albany NY). 2014;6:820-34.

S5. Chen D, Steele AD, Lindquist S, Guarente L. Increase in activity during calorie restriction requires Sirt1. Science. 2005;310:1641.

S6. Collins MT, Marcucci G, Anders HJ, Beltrami G, Cauley JA, Ebeling PR, et al. Skeletal and extraskeletal disorders of biomineralization. Nat Rev Endocrinol. 2022;18:473-89.

S7. Baron R, Rawadi G. Targeting the Wnt/beta-catenin pathway to regulate bone formation in the adult skeleton. Endocrinology. 2007;148:2635-43.

S8. Maeda K, Kobayashi Y, Koide M, Uehara S, Okamoto M, Ishihara A, et al. The Regulation of Bone Metabolism and Disorders by Wnt Signaling. Int J Mol Sci. 2019;20.

S9. Hayat R, Manzoor M, Hussain A. Wnt signaling pathway: A comprehensive review. Cell Biol Int. 2022;46:863-77.

S10. Zhang Q, Pan Y, Ji J, Xu Y, Zhang Q, Qin L. Roles and action mechanisms of WNT4 in cell differentiation and human diseases: a review. Cell Death Discov. 2021;7:287.

S11. Gozo MC, Aspuria PJ, Cheon DJ, Walts AE, Berel D, Miura N, et al. Foxc2 induces Wnt4 and Bmp4 expression during muscle regeneration and osteogenesis. Cell Death Differ. 2013;20:1031-42.

S12. Yu B, Chang J, Liu Y, Li J, Kevork K, Al-Hezaimi K, et al. Wnt4 signaling prevents skeletal aging and inflammation by inhibiting nuclear factor-kappaB. Nat Med. 2014;20:1009-17. doi:10.1038/nm.3586

S13. Viguet-Carrin S, Garnero P, Delmas PD. The role of collagen in bone strength. Osteoporos Int. 2006;17:319-36.

S14. Robinson MM, Dasari S, Konopka AR, Johnson ML, Manjunatha S, Esponda RR, et al. Enhanced Protein Translation Underlies Improved Metabolic and Physical Adaptations to Different Exercise Training Modes in Young and Old Humans. Cell Metab. 2017;25:581-92.

S15. Kirk B, Zanker J, Duque G. Osteosarcopenia: epidemiology, diagnosis, and treatment-facts and numbers. J Cachexia Sarcopenia Muscle. 2020;11:609-18.

S16. Gao Q, Mei F, Shang Y, Hu K, Chen F, Zhao L, et al. Global prevalence of sarcopenic obesity in older adults: A systematic review and meta-analysis. Clin Nutr. 2021;40:4633-41.

S17. Wang Z, Gerstein M, Snyder M. RNA-Seq: a revolutionary tool for transcriptomics. Nat Rev Genet. 2009;10:57-63.

S18. Moro T, Brightwell CR, Volpi E, Rasmussen BB, Fry CS. Resistance exercise training promotes fiber type-specific myonuclear adaptations in older adults. J Appl Physiol (1985). 2020;128:795-804.

S19. Harber MP, Konopka AR, Douglass MD, Minchev K, Kaminsky LA, Trappe TA, et al. Aerobic exercise training improves whole muscle and single myofiber size and function in older women. Am J Physiol Regul Integr Comp Physiol. 2009;297:R1452-9.

S20. Grossmann M, Jayasena CN, Anawalt BD. Approach to the Patient: The Evaluation and Management of Men >/=50 Years With Low Serum Testosterone Concentration. J Clin Endocrinol Metab. 2023;108:e871-e84.
