# Supplementary material for: Testosterone Modulation of Muscle Transcriptomic Profile During Lifestyle Therapy in Older Men with Obesity and Hypogonadism
Source: J Cachexia Sarcopenia Muscle. 2025 Jan 27;16(1):e13697. doi: 10.1002/jcsm.13697 (PMC11924803; doi:10.1002/jcsm.13697)
Supplement: Supplementary file 2 — Data S1 Supplementary Information. [file JCSM-16-e13697-s001.pdf]

Compare
New Compare

Institutional Review Board for Baylor College of Medicine and Affiliated Hospitals

Protocol Number: **H-35267**
Status: Approved
Initial Submit Date: 7/2/2014
Approval Period: 12/20/2023 - 1/15/2025

Section Aa: Title & PI

A1. Main Title

TESTOSTERONE REPLACEMENT TO AUGMENT LIFESTYLE THERAPY IN OBESE OLDER VETERANS

A2. Principal Investigator

Name:	DENNIS VILLAREAL	Phone:	713-798-3185
Id:	184416	Fax:	713-798-4585
Department:	MEDICINE: ENDOCRINOLOGY	Email:	dennis.villareal@bcm.edu
Center:		Mail Stn:	BCM185

A3. Administrative Contact

Name:	HUI DONG	Phone:	7137983185
Id:	180783	Fax:	
		Email:	hdong@bcm.edu
		Mail Stn:	BCM285

A3a. Financial Conflict of Interest

Does any member of study personnel (Investigator (including investigator's spouse and/or dependent children)) that are involved in the design, conduct, or reporting of the research have a Significant Financial Interest (SFI) that would reasonably appear to be affected by the research for which funding is sought and/or associated with an entity/business that would reasonably appear to be affected by the research?

No

Section Ab: General Information

A4. Co-Investigators

Name:	BENNY KAIPPARETTU	Phone:	7137986506
Id:	152363	Fax:	
Department:	MOLECULAR & HUMAN GENETICS	Email:	kaippare@bcm.edu
Center:		Mail Stn:	BCM225
Name:	BRYAN JIANG	Phone:	713-798-0190
Id:	177335	Fax:	
Department:	MEDICINE: ENDOCRINOLOGY	Email:	bjiang@bcm.tmc.edu
Center:		Mail Stn:	BCM620
Name:	REINA VILLAREAL	Phone:	713-798-3185
Id:	184419	Fax:	713-798-4585
Department:	MEDICINE: ENDOCRINOLOGY	Email:	reina.villareal@bcm.edu
Center:		Mail Stn:	BCM185
Name:	JAVAD RAZJOUYAN	Phone:	7137987928

Id: 192641
Department: MEDICINE: HEALTH SRVCS RESEARCH
Center:
Fax: 713 7981499
Email: razjouya@bcm.edu
Mail Stn: BCM288

Name: MARIA LIZA NAVA
Id: 196882
Department: MEDICINE: ENDOCRINOLOGY
Center:
Phone: 713-794-7156
Fax: 713-798-8764
Email: MariaLiza.Nava@bcm.edu
Mail Stn: BCM185

Name: ADRIAN GONZALEZ GIL
Id: 238408
Department: MEDICINE: RESIDENTS ONLY
Center:
Phone:
Fax:
Email: u238408@bcm.edu
Mail Stn:

Name: TAGARI SAMANTA
Id: 241680
Department: MOLECULAR & HUMAN GENETICS
Center:
Phone:
Fax:
Email: u241680@bcm.edu
Mail Stn:

Name: VIOLA VIOLA
Id: 247792
Department: MEDICINE: ENDOCRINOLOGY
Center:
Phone:
Fax:
Email: u247792@bcm.edu
Mail Stn:

Name: CLIFFORD QUALLS
Id: Non-Baylor
Institution: Biomedical Research Institute of New Mexico
Address: 1501 San Pedro Drive, Albuquerque, NM 87108
Phone: (505) 249-3991
Fax:
Email: Clifford.Qualls@va.gov

A5. Funding Source:

Organization: VA CENTRAL OFFICE, RR&D

A6a. Institution(s) where work will be performed:

BCM: Baylor College of Medicine
Michael E. DeBakey Veterans Affairs Medical Center

A6b. Research conducted outside of the United States:

Country:
Facility/Institution:
Contact/Investigator:
Phone Number:

If documentation of assurances has not been sent to the Office of Research, please explain:

A7. Research Category:

A8. Therapeutic Intent

Does this trial have therapeutic intent?
Yes

A9. ClinicalTrials.gov Registration

Does this protocol/trial require registration on ClinicalTrials.gov due to it: meeting the definition of an Applicable Clinical Trial, being required under the terms and conditions of an award, or being proposed to be published in ICMJE journals?
Yes

Who will be responsible for registering and maintaining the registration of this Applicable Clinical Trial?
The BCM PI will register the trial because either:

- the trial is BCM PI-initiated,

- BCM is the lead site of this multicenter trial, or,
- the industry sponsor has instructed the BCM PI to register the trial, or,
- registration of this trail is required as a term and condition of the reward by the funding agency.

ClinicalTrials.gov Identifier:
NCT02367105

Section B: Exempt Request

B. Exempt From IRB Review

Not Applicable

Section C: Background Information

Obesity among American veterans is highly prevalent and even more so among veterans using VA medical facilities. As previously discussed, as much as 40% of obese veterans are over 65 years old and expected to increase.15 Decreased muscle mass with aging and the need to carry extra mass due to obesity make it particularly difficult for obese older adults to function independently and results in frailty leading to increased nursing home admissions and increased morbidity and mortality. Failure to assist veterans in managing weight and sedentary lifestyle affects current treatment and future demand for VA health care services. For instance, a study among veterans showed that advancing frailty is associated with higher hospitalization and post-hospitalization cost, as well as higher post-discharged institutionalization and 30-day readmission. Given the high prevalence of obesity among veterans, it is likely that obesity contributed in a major way to frailty in these subjects. However, there is little evidence-based data to guide treatment of obesity in older adults and appropriate management of obese older adults is controversial. The MOVE (Managing Overweight/ Obese Veterans) program does not have any guidelines for eligible veterans if they are 70 or older. As discussed above, weight loss can be beneficial in obese older adults by improving function and quality of life or harmful by causing a decrease in muscle mass and bone mass. Data from our group showed that exercise only attenuates but not totally prevent the weight loss-induced reduction of muscle mass and bone mass. These findings, superimposed on age-related declines in muscle mass and bone mass, suggest that loss of muscle mass and loss of bone with voluntary weight loss might be harmful by further decreasing the reserve capacity of these tissues. Preservation of muscle and bone during voluntary weight loss in older adults may be important in keeping them above the threshold for risk for disability and fracture. Considering the high prevalence of testosterone deficiency in our population of male obese older adults, we propose that the restoration of anabolic hormone levels through testosterone replacement will preserve muscle mass and bone mass and reverse frailty in obese older adults undergoing lifestyle therapy. The novel data generated from this proposed RCT will potentially impact the future recommendations for the standard of care in this rapidly increasing segment of the aging male veteran population

Section D: Purpose and Objectives

Primary aims Aim 1. Determine the effect of lifestyle therapy (diet-induced weight loss and exercise training) + testosterone replacement (LT+Test) compared to lifestyle therapy + testosterone placebo (LT+Pbo) on physical function Aim 2: Determine the effect of LT+Test compared to LT+Pbo on lean body mass and muscle quality. Aim 3: Determine the effect of LT+Test compared to LT+Pbo on bone mineral density (BMD) and bone quality.

Secondary aims Determine the effect of LT+Test compared to LT+Pbo on local catabolic and anabolic factors in skeletal muscle. Other secondary aims include determining the effect of LT+Test compared to LT+Pbo on cardiodiovascular risk factors, cognition, mood, and quality of life.

The data to be obtained will not be used for future research and will be used only for purposes outlined in this protocol.

Section E: Protocol Risks/Subjects

E1. Risk Category

Category 2: Research involving greater than minimal risk, but presenting the prospect of direct benefit to the individual subjects.

E2. Subjects

Gender:
Male

Age:
Geriatric (65+ yrs)

Ethnicity:
All Ethnicities

Primary Language:
English

Groups to be recruited will include:
Patients

Which if any of the following vulnerable populations will be recruited as subjects?

Vulnerable populations require special protections. How will you obtain informed consent, protect subject confidentiality, and prevent undue coercion?

E3. Pregnant woman/fetus

Will pregnant women and/or fetuses (as described in 45 CFR 46 Subpart B) be enrolled in the research?
No

E4. Neonates

Will neonates of uncertain viability or nonviable neonates (as described in 45 CFR 46 Subpart B) be enrolled in the research?
No

E5. Children

Will children be enrolled in the research?
No

Section F: Design/Procedure

F1. Design

Select one category that most adequately describes your research:
z.r) Randomized, Efficacy Study -- Surgical Techniques/Interventions

Discuss the research design including but not limited to such issues as: probability of group assignment, potential for subject to be randomized to placebo group, use of control subjects, etc.
This will be a randomized, comparative efficacy, double-blind, placebo-controlled (for testosterone) trial of the effects of lifestyle therapy (10% diet-induced weight loss and exercise training) + testosterone replacement versus lifestyle therapy without testosterone replacement on physical function, lean body mass, BMD and bone quality, and local and systemic catabolic and anabolic factors that mediate these changes in obese older men

Inclusion Criteria:
Subjects will be older (65-85 yr.) 2). Obese men (BMI 30 kg/m2 or greater) with low testosterone (less than 300 ng/dL) as defined by the Endocrine Society. To confirm biochemical hypogonadism, serum testosterone concentrations will be measured on two separate mornings and shown to be consistently less than 300 ng/dL. 3). All subjects will be mild to moderately frail, based on screening tests described below. 4). All subjects must have stable weight (~not less than or more than 2 kg) during the last 6 months. 5). Sedentary (regular exercise less than 1 h/week or less than 2 x/week for the last 6 months). As in our previous studies (e.g. Villareal et al, Obes Res 2004, New Engl J Med 2011), the screening for physical frailty will include assessments of physical function using the modified physical performance test (PPT), aerobic capacity (peak oxygen consumption, VO2peak) using the graded exercise test, and basic and activities of daily living using the functional status questionnaire.

Assessment of physical frailty. To be eligible for the study, individuals have to meet criteria for mild to moderate frailty based on a modified Physical Performance Test (PPT) score of 18¿31 (score range 0-36). Low peak aerobic power (VO2peak) of 11 to 18 ml/min per kg of body weight and/or 3) self-reported difficulty or need for assistance with 1 basic activity of daily living or up to 2 instrumental activities of daily living will also provide support for frailty. Subjects will be excluded from this study if they are considered too physically frail (e.g. have a score of less than 18 on the PPT and/or VO2peak below 10 ml/kg/min).

Exclusion Criteria:
Any major chronic diseases, or any condition that would interfere with exercise or dietary restriction, in which exercise or dietary restriction are contraindicated, or that would interfere with interpretation of results. Examples include, but are not

limited to: a) cardiopulmonary disease (e.g., recent MI, unstable angina, stroke etc.) or unstable disease (e.g., CHF). b) severe orthopedic/musculoskeletal or neuromuscular impairments; c) visual or hearing impairments ; d) cognitive impairment (Mini-Mental State Exam Score less than 24); and e) current use of bone acting drugs (e.g. estrogen, androgen containing compounds, raloxifene, calcitonin, parathyroid hormone during the past six months or biphosphonates during the last two years) and f) uncontrolled diabetes (i.e. fasting blood glucose more than140 mg/dl and/or HbA1c greater than 9.5%). 2. Any contraindications to testosterone supplementation:69 a) history of prostate cancer or breast cancer, b) history of testicular disease, c) untreated sleep apnea, d) hematocrit of more than 50%, and e) prostate-related findings of a palpable nodule on exam, a serum PSA of 4.0 ng/ml or greater, International Prostate Symptom Score more than 19. 3. Osteoporosis or a BMD T-score of -2.5 in the lumbar spine, total hip as well as those patients with a history of osteoporosis-related fracture (spine, hip or wrist), and 4. Personal or family history of venous thromboembolism

F2. Procedure

A. Screening tests- to determine whether volunteers are eligible to participate they will undergo a medical history, physical examination, blood tests, physical function tests, DXA (bone scan), electrocardiogram (EKG), and graded exercise test. In some volunteers, if there is a question if there is metal in their eye (e.g. welders) they will also undergo a skull x-ray as part of safety measure prior to proceeding with MRI.

B. Baseline and follow-up tests- if they are eligible to participate and choose to do so, they will undergo a series of tests and procedures: a. Body Composition assessments Dual energy x-ray absorptiometry (DXA). The amount of fat, muscle, and bone in your body will be measured by DXA and Magnetic Resonance Imaging (MRI). Fat in the abdomen and thighs will be measured by MRI. Also, brain images will be collected. Quantitative computed tomography (QCT) scan. This is another form of bone density testing which measures volumetric bone mineral density and cross sectional bone dimensions of the leg. High resolution quantitative computed tomography (HR-pQCT). This machine is able to assess the 3-dimensional bone microarchitecture and volumetric bone mineral density of the leg and forearm. Coupled with computer-based finite element analysis (FEA) modeling, it will also provide a newfound approach to assess bone strength. Waist circumference will be measured with a tape measure. MRI, QCT and HR-pQCT scan will be done at baseline and 6 months. DXA scan will be done at baseline, 3 months and 6 months. b. Blood tests: Blood will be drawn for routine chemistries (blood count and electrolytes), blood sugar, cholesterol, hormones (including testosterone), prostate specific antigen, and markers of bone function and inflammation. These tests will be performed at baseline and after 3 and 6 months. Additional blood test as needed every two weeks will also be performed after any medication change. c. Physical function tests: The Physical Performance Test will evaluate ability to do activities such as walking, climbing steps, and picking up small objects such as a coin. Flexibility testing will be done by checking range of motion of your joints. Muscle strength will be tested by having them use their arms or legs to push and pull as hard as possible against a special machine, and stand up from a kneeling position and sit down from a standing position. Balance will be determined by having a tester examine their ability to stand with your feet in different positions, and walk an obstacle course. Endurance will be measured by how quickly and how far they can walk within a specific amount of time. These tests will be done at baseline, after 3-months and at the end of the study. d. Questionnaires: The SF-36 will ask you how you feel about your quality of life. The Functional Status Questionnaire will ask about things that they all need to do as a part of our daily lives. The Impact of Weight on Quality of Life Questionnaire- Lite will evaluate the effect of excess weight on their quality of life. The psychometric tests and mood scale will assess cognition and mood. The physical activity questionnaire will evaluate their level of physical activity. International Index of Erectile Function (IIEF) questionnaire will assess sexual function. These will be completed at baseline and at the end of the study. e. Measurement of aerobic capacity: They will be asked to walk on a treadmill while breathing through a mouthpiece, and the amount of oxygen they consume will be measured. This test evaluates the ability of their heart to provide your muscles with oxygen. This test will be performed at baseline and at the end of the study. f. Diet evaluation: They will meet with a dietitian for about ½ hour. The dietitian will ask them to measure and write down everything that they eat and drink for 3 days. At the end of the 3 days, they will meet with the dietitian again to review your food diary. This will be done at baseline, 3 months, and at the end of the study.

Muscle studies: These tests are optional and are not required in order to take part in the overall study. These tests will be done at baseline and after 6 months. We will obtain by biopsy samples of muscle tissue from your thigh. This involves numbing their skin with local anesthesia (by injecting 2% xylocaine), making a small (approx. the size of the dotted line ------; ½ cm) incision on the thigh for the muscle biopsy, removing a small (approx. 1/15th of an ounce) piece of muscle tissue. The incision is then closed with a piece of sterile tape. They will be given instructions on how to take care of the biopsy site to prevent any complications.

C. Interventions

After the medical screening and baseline assessments are complete, they will be randomly assigned to receive either: 1) Lifestyle therapy (weight loss and exercise) + testosterone 2) Lifestyle therapy (weight loss and exercise) + placebo

All participants will participate in lifestyle therapy (standard first step approach for treating obesity) consisting of a weight management program and exercise training program. They will receive, under the care of a registered dietitian, a special diet and attend group behavior modification sessions lead by the dietitian. These sessions will include classes on topics such as keeping track of what they eat, using food diaries, stress management, problem-solving, social support, changing problem behaviors, and identifying reasons or cues for eating. These sessions will last 75-90 min, which will be held weekly. They will be asked to lose at least 10% of their body weight during the intervention. In addition, they will participate in the exercise training sessions on 3 nonconsecutive days for approximately 60-90 minutes at our exercise facility. Each session will be led by a physical therapist/exercise trainer and will include exercise to improve flexibility, strength, endurance, and balance. Due to unexpected changes in circumstances, interruption of regular exercise and/or participation

in diet classes may occur. Such subjects may be reintegrated into the exercise program. The exercise physiologist will reassess their maximal strength and aerobic capacity to establish a new exercise prescription.

The testosterone or placebo will be given in the form of a gel to be applied once daily in the morning to clean, dry, intact skin of their shoulders and upper arms. They will be given detailed instructions regarding the proper administration of the study medication. This may require an adjustment in dose if necessary depending on the results of the blood tests that monitor safety or adequacy of therapy. To monitor safety or any necessary adjustment in dose, one of the study physicians will know if they are on the drug or the placebo, but neither the participant nor the rest of the study team will know which medication they are taking as they will be identical in appearance. However, this information is available in case of an emergency. They will continue to take this medication for up to 6 months.

The testosterone gel will be Androgel 1.62%, which is available in the MEDVAMC formulary for VA patients and approved for replacement therapy in males with deficiency of endogenous testosterone (hypogonadism). As per the package insert, starting dose of Androgel 1.62% will be 40.5 mg of testosterone (single 40.5 mg packet) once daily. To verify that serum testosterone levels are in the target range, serum testosterone will be determined two weeks after starting application. If testosterone concentration is less than 350 mg/dL, the unblinded study physician will increase the testosterone dose to 60.75 mg (one 40.5 mg packet plus one 20.25 mg packet daily) daily. If testosterone concentration is greater than 750 mg/dl, the unblinded study physician will decrease the dose by 20.25 mg (one 20.25 mg packet). Subsequent dosage adjustments will also be based on symptoms and the occurrence of side effects as performed by the same unblinded study physician who will not be involved in baseline or follow-up testing. To maintain blinding, the physician making the dosage adjustments will direct that a participant in the placebo group be treated similarly.

The health records of subjects will be flagged.

Muscle biopsy is needed to address secondary aim ¿Determine the effect of lifestyle therapy + testosterone (LT + Test) compared to lifestyle therapy + placebo (LT + Pbo) on local catabolic and anabolic factors in skeletal muscle¿. We hypothesize that LT+Test will cause a greater reduction in intramuscular proinflammatory cytokines than LT+Pbo. This hypothesis will be tested by comparing changes in gene and protein expression of cytokines such as TNF-á, IL-6, IGF-1 and muscle specific isoform (mechano growth factor) in muscle tissue samples between the treatment groups.

Expression of genes and proteins involved in inflammation, anabolism, and catabolism. Real time RT-PCR. Total RNA will be isolated from muscle tissue biopsies according to the manufacturer¿s protocol. Briefly, frozen muscle samples will be disrupted on ice using a tissue homogenizer. Chloroform will be added (1/10 original volume), the homogenate mixed, and samples centrifuged to separate the aqueous and organic phases, which will remove DNA and protein from the RNA-containing aqueous phase. RNA will then be precipitated from the aqueous phase by the addition of isopropanol and centrifugation. The resulting RNA pellet will be washed in 75% ethanol, dried, and resuspended in RNAse-free water. RNA quantity and purity will be determined spectrophotometrically and bioanalyzer. Gene expression will be quantified by using real-time RT-PCR. . Samples will be assayed in triplicate. Triplicates wherein the variation is greater than 10% from the average of the three will be discarded and re-run. The expression of the following genes will include: (1) TNF-á, IL-6 (pro-inflammatory cytokines, catabolism), and (2), MGF and IGF-1 (anabolism,) as well as also other genes involved in inflammation, anabolism, and catabolism. Arbitrary units of target mRNA will be corrected to levels of housekeeping gene. The expression of CD68, a marker macrophage infiltration, will also be determined to evaluate the relative number of resident macrophages. . We may also use micro-array or RNA-seq based RNA expression of relevant genes in tissue samples to help understand the mechanisms or mediators for the effects of our testosterone replacement and lifestyle therapy in obese older veterans. Western blotting: Frozen muscle tissue will be homogenized in ice cold buffer containing: 50 mM Tris- (ph7.4), 1% NP-40, 0.25% sodium deoxycholate, 150 mM NaCl, 1 mM EDTA, 1 mM PMSF, protease inhibitor cocktail (Sigma Chemical Co.), and 2 mg/ml glycerophosphate. Samples will be incubated on ice for 30 minutes and then centrifuged for 15 min at 15,000 X G. The supernatant will then be collected and the protein concentration will be determined using the Lowry method. Samples will be diluted to a common total protein concentration and prepared in Laemmli sample buffer containing 100 mM dithiothreitol, denatured at 100C for 5 minutes and subjected to SDS-PAGE. Protein will be transferred from the gel to nitrocellulose membrane. Membranes will be blocked for 1-1.5 hours in TBS containing 0.1% tween 20 (TBS-T) and 5% non-fat milk. Blots will be probed using commercially available antibodies such as rabbit anti-human IL-6, rabbit anti-human TNF-á, rabbit anti-human IGF-1, anti-human actin, overnight at 4°C in TBS-T /1% milk. Blots will be washed in TBS-T and then incubated with donkey anti-rabbit HRP-labeled IgG secondary antibody for 1 hr at room temperature. The muscle samples will also be used to measure changes in mitochondrial function in response to lifestyle interventions + testosterone/placebo.

Section G: Sample Size/Data Analysis

G1. Sample Size

How many subjects (or specimens, or charts) will be used in this study?

Local: 500 Worldwide: 500

Please indicate why you chose the sample size proposed:

Based on our power calculations below, 84 subjects randomized are needed to have strong power to detect significant differences in our primary outcomes. Because of the extensive screening process that are needed to determine eligibility, we anticipate that up to 500 subjects will need to be consented to come up with 84 eligible subjects to be randomized for

the study. The computations that follow are based on a target sample size of 41 randomized subjects in each of the two study groups. All computations are based on two-sided tests at the 0.05 level of significance. It is assumed that 20% of subjects will drop out. Physical performance test score (PPT): Pilot data from our previous and ongoing studies showed that the change in PPT in response to 6 months of LT (weight loss and exercise training) ranged from 2.6 ± 2.5 to 4.7 ± 2.43 while the change in PPT in response to weight loss alone was 1.3 ± 2.0 and in response to exercise alone was 2.3 ± 1.2. We expect that LT+Test will cause a greater improvement in PPT than LT+Pbo. Accordingly, based on the largest pooled SD from all these pilot data (i.e. 2.5), we will be able to detect differences in the means (changes in response to LT+Test vs. LT+Pbo) of 1.8 in PPT (e.g., 3.6 vs. 5.4) with a power of 0.89, consistent with reversal of frailty (e.g. PPT > 32). The power will be 0.94 if the difference is as large as 2.4 (e.g. 3.6 vs. 6.0). Previous studies showed improvement in physical function (e.g. an increase in PPT score of 1.8 ± 2.2 in frail elderly men) in response to testosterone replacement alone. Fat free mass (FFM): Meta analytic results from the literature indicate a projected weight loss-induced loss of 3.0 + 0.3 (standard error) kg of FFM, with reported SDs in other reports of around 2.1. Indeed, our own pilot data also showed that weight loss alone was associated with a loss of FFM of 3.5 ± 2.1, whereas lifestyle therapy (weight loss + exercise) was associated with a loss of FFM of 1.8 ± 1.5 kg Assuming the largest pooled SD among these data (i.e. 2.1), the power to compare preservation of FFM in response to LT+Test with FFM loss in response to LT+Pbo is 0.89 (e.g. 0.0 kg vs. -1.8 kg). The power will be 0.98 if the addition of testosterone replacement to lifestyle therapy (LT+Test) will modestly increase FFM compared to a decrease in response to LT+Pbo (e.g. 0.5 kg vs. -1.8 kg). Previous studies showed an increase in FFM of 1.6 - 3.7 kg in response to testosterone replacement therapy.11 Hip bone mineral density (BMD): Previous data indicated that diet-induced weight loss interventions are associated with reductions in hip BMD of 2.6 ± 2.1% (Ryan et al), 4.2 ± 1.2%, (Ricci et al.) and 4.0% (Jensen et al.) where SD was not presented in the last paper. Indeed, our own pilot data (Villareal et al) showed that exercise was unable to prevent this weight loss-induced reduction in hip BMD (2.4 ± 2.1%). Assuming the largest pooled SD (2.1), the power to compare preservation of hip BMD in response to LT+Test with BMD loss in response to LT+Pbo is 0.94 (e.g. 0.0 vs. -2.4%). Assuming that LT+Test will attenuate the BMD loss induced by LT+Pbo, the power will still be 0.70 (e.g. -1.0 vs. -2.4%). Previous studies showed an increase in BMD of 1.4 - 3.5 % in response to testosterone replacement therapy in hypogonadal men. Regarding the effect of LT+Test on muscle cytokines and growth factors, our preliminary data in obese older adults suggested a between group difference of .53 + .23 for IL-6 mRNA, 1.7 + .4 for TNF-á , and 3.8 + 1.8 for MGF mRNA. Thus, we will have ample power (0.99) to detect between group differences. Assuming that the addition of testosterone replacement to lifestyle therapy (LT+Test) will be half as effective (e.g. between group difference of 0.27, 0.9, and 1.9) and using the same SDs (i.e. 0.23, 0.4, and 2.2), calculations would still yield adequate power (0.99, >0.99, and 0.98 respectively).

G2. Data Analysis

Provide a description of your plan for data analysis. State the types of comparisons you plan (e.g. comparison of means, comparison of proportions, regressions, analysis of variance). Which is the PRIMARY comparison/analysis? How will the analyses proposed relate to the primary purposes of your study?

Intention-to-treat analyses will be performed by analyzing data from all participants originally randomized. The longitudinal analysis of all outcomes will be performed using a random effects mixed model repeated measures analysis of covariance in which lifestyle therapy + testosterone replacement therapy (LT+Test) and lifestyle therapy + testosterone placebo (LT+Pbo) constitute the primary factors to be evaluated and potential covariates may include baseline values, years of education (for cognitive measures), and other measures of clinical status as appropriate. Mixed model analyses use all the available data and deals easily with missing data due to missed visits and subject drop-out.116 The mixed model approach (PROC MIXED in SAS) we will employ fits a linear model including a group-time interaction effect (the change over visits is different in the two intervention groups) as well as potential covariates, and allowing for correlation between repeat measurements on the same subject. Prior to performing the longitudinal analyses, more routine preliminary analyses will be performed. These include standard tests aimed at ensuring that both outcome measures and continuous covariates are similar across groups at baseline. Categorical data analyses with Fisher¿s exact test will provide information about between group balance with respect to dichotomous covariates. In all analyses, careful attention will be given to ensuring that the conditions that are required of a particular statistical method are satisfied. Thus, we will assess the normality of regression residuals and ensure the homogeneity of variances by examining residuals after analyses of variance and covariance models are fit. When required assumptions are not satisfied, data transformations will be explored and utilized as appropriate. If transformations that yield the required assumptions cannot be found, it may be necessary to analyze some variables semi-parametrically using the ranks of the data. We will also perform multiple imputation analyses for missing fitness data. Our overarching hypothesis across aims (theoretical model) is that a multifactorial intervention by means of LT+Test will be the most effective approach for reversing sarcopenic obesity and frailty in obese older adults, as mediated by their additive effects in suppressing chronic inflammation and stimulating anabolism. Therefore, to test this central hypothesis in an integrated manner, we will also use partial correlation analyses and stepwise multiple regression analyses to determine which of the body composition factors or the mechanistic skeletal muscle factors are the most important mediators for the observed changes in overall physical function (PPT score). We will use partial correlation and multiple regression to control for possible overlap among the independent variables, and, thus, sorting out the important contributions of the preservation of lean mass and of the different mechanistic skeletal muscle factors (e.g., changes in TNF-á, IL-6 mRNA and protein) on the magnitude of improvement in overall PPT score (primary functional outcome).

Section H: Potential Risks/Discomforts

H1. Potential Risks/Discomforts

Describe and assess any potential risks/discomforts; (physical, psychological, social, legal, or other) and assess the likelihood and seriousness of such risks:

We anticipate no psychological, social or legal risks beyond those of participation in health-related research in general. The potential risks of this study are small. The program of weight loss treatment presents few risks to subjects. The potential risks of the study are primarily associated with the testing measures, exercise testing, and testosterone therapy as described in the next paragraphs:

Risks of testing procedures The general data collection procedures involve patient interviews and questionnaires that are time- consuming (60-90 minutes) and may result in fatigue or aggravation. In addition, some questions may touch on emotionally sensitive issues that could cause anxiety or other forms of emotional stress. There is a small risk of a hematoma, and an extremely small risk of infection associated with insertion of needles for blood withdrawal. An experienced technician, using aseptic technique will minimize these risks. The muscle strength testing protocol involves isometric and isokinetic exercise against resistance. These exercises are brief and simulate those performed in a standard neuromuscular exam. The risk of these evaluations is therefore minimal. However, an occasional patient might experience muscle fatigue or soreness after testing. The gait and balance measures could result in a fall. The DXA, pQCT and HR-pQCT will involve exposure to radiation. The amount of radiation averaged over the entire human body is equivalent to 6.5 mrem (0.065 mSv). This is equivalent <1% of the allowable annual dose to radiation workers (e.g. X-ray technicians) and < 3% of the amount of natural background radiation exposure of people in the Albuquerque, NM area receive annually. It is of little consequence when compared to other everyday exposure risks. With the MRI exam, some patients experience slight tingling or tapping sensations in the arms or legs. This occurs in 2-4% of patients and does not cause any damage to tissue. The muscle biopsy may result in some mild discomfort or infection. To minimize the discomfort a small amount (2-3 ml) of local anesthetic (2% lidocaine) is injected at the incision site just prior to the biopsy. Bleeding at the incision is minimized by applying direct pressure to the incision. The muscle sample is removed from a superficial portion of the vastus lateralis muscle where the risk of cutting a large artery or nerve is minimal. Our group has obtained over 1050 muscle biopsies in study participants ranging in age from 24 yr to 92 yr, the majority of whom have been elderly. The only complications have been one mild hematoma and some soreness in the thigh for several days following a biopsy. The risks of the muscle biopsy procedure include discomfort, bleeding or infection at the site of insertion. Exercise testing, using an appropriate protocol in carefully selected patients is associated with serious complications in fewer than 1 in 5,000 patients. In a review of complications of 170,000 exercise tests conducted in 73 medical centers in the USA, Rochmis and Blackburn (JAMA 271:1060, 1971) reported a mortality rate of one per 10,000 patients tests; these were patients who were known to have or suspected of having ischemic heart disease. Repeated exercise testing, when properly monitored under the supervision of an experienced physician, does not appreciably increase the risk to the patient. During the 11 years of the Program Project "Physiological Adaptations to Exercise" and during our 5-year Claude Pepper Older Americans Independence Center (OAIC) project, repeated maximal exercise tests have been performed on more than 650 people age 60-96 years enrolled in research projects without any significant problems. Complications can include severe angina, acute myocardial infarction, serious arrhythmias, and death. Less serious problems include fatigue, dyspnea, and lightheadedness.

Risks of exercise training The potential risks of exercise training include development of ventricular arrhythmia, myocardial infarction, cardiac arrest, and death. Haskell (Circulation 57:220, 1978) has reviewed the experience of 30 cardiac rehabilitation programs in North America and reported information on 13,750 participants, who accumulated 1,629,634 patient hours of supervised exercise. A total of 50 cardiac arrests were observed during exercise, 42 of which were successfully resuscitated, while 8 were fatal. Available information from randomized trials of exercise training in the secondary prevention of ischemic heart disease suggest that the mortality rate is lower in ISCHD patients who exercise than in comparable patients who remain sedentary (Ann Clin Res 4: Suppl 9, 1972; Acta Med Scand Suppl 599, 1976, Am J Cardiol 48:39, 1981) when mortality from these studies are combined, the average total percent mortality for the exercising groups is 8.9%, versus 12.4% for the controls ($p < 0.05$). The above information regarding complications of exercise testing and training in CAD patients is reviewed because no statistical information is available regarding the complication rates during exercise in healthy people without evidence of ISCHD. It seems reasonable to assume that people without evidence of clinical ISCHD will have a lower incidence of cardiovascular complication during medically supervised exercise testing and training than patients with ISCHD. In our OAIC studies of exercise in frail older adults we have not encountered any significant problems with the exercise testing or training (n=150). The exercise training could cause problems due to injury of tendons, ligaments, joints, or muscles. There is also the risk of a fall during exercise training, with related injury. During our current study we have not had any injuries related to the exercises, or any injurious falls. In a previous study of 115 frail elderly men and women who participated in a 9- month exercise training program, only 2 individuals had tendon injuries, and there were no other exercise-related injuries.100,107 The exercise facility is equipped with a defibrillator and all appropriate emergency medications. Subjects with known or suspected serious heart disease will not be accepted into the study. Apart from non-participation there is no specific alternative to this study. Subjects may choose to participate in other clinical research at our Center.

Risks of testosterone replacement therapy (TRT) There will be risk associated with testosterone therapy such as an increase in hemoglobin/hematocrit above a certain level may be associated with hyperviscosity and bad outcomes especially in the elderly such as exacerbation of vascular disease in the peripheral circulation, the coronaries or the cerebrovascular circulation. The association between TRT and the increased risk of major adverse cardiovascular events is still under discussion. Although still controversial, the Food and Drug Administration has recently advised health care professionals to make patients aware of possible increased risk of heart attack and stroke in patients on treatment with testosterone. These subjects may also develop prostate enlargement and potential for increase in urinary problems including lower urinary tract symptoms (LUTS), urinary retention and potentially stimulating the development of prostate cancer, although a definite association with the latter has not been proven. Hepatotoxicity, benign or malignant hepatic tumors, cholestasis have been reported with the oral form of testosterone therapy but do not appear to be associated with intramuscular or transdermal preparation.61 There is also the potential of developing gynecomastia, leg edema, acne and exacerbation of pre-existing sleep-apnea as well as very mild changes in lipid panel. Other risks are mostly associated with

the injections such as pain, hematoma and the very small risk for infection at the site of injection or the site of fat biopsy. Other rare risksalso include priapism (erection more than 4 hours), and behavioral change such as aggressiveness and irritability. In addition, other members of the household who get exposed could develop masculinizing signs and symptoms.

To minimize thisrisks, patients will be carefully advised of the following instructions: - Read the Medication Guide before starting thetestosterone gel therapy and to reread it each time the prescription is renewed. The testosterone gel should be applied and used appropriately to maximize the benefits and to minimize the risk of secondary exposure to household members, particularly females and children. Children and women would avoid contact with unwashed or unclothed application sites of men using the testosterone gel. Patients using the gel should apply the product as directed and strictly adhere to the following: Wash hands with soap and water immediately after application. Cover the application site with clothing after the gel has dried. Wash the application site thoroughly with soap and water prior to any situation where skin-to-skin contact of the application site with another person is anticipated. In the event that unwashed or unclothed skin to which the testosterone gel has been applied comes in contact with the skin of another person, the general area of contact on the other person should be washed with soap and water as soon as possible.

There are risks of stress, emotional distress, inconvenience and possible loss of privacy and confidentiality.

H2. Data and safety monitoring plan

Do the study activities impart greater than minimal risk to subjects?

Yes

NOTE: The answer to the questions in H2 requires the completion of the form: 'Section H â€“ Data and Safety Monitoring Plan' as an attachment in Section S.

H3. Coordination of information among sites for multi-site research

Is the BCM Principal Investigator acting as the SPONSOR-INVESTIGATOR for this multi-site research?

No or Not Applicable

Is BCM the COORDINATING CENTER for this multi-site research?

No or Not Applicable

Section I: Potential Benefits

Describe potential benefit(s) to be gained by the individual subject as a result of participating in the planned work.

All subjects will receive the benefits of exercise and weight loss therapy for obesity. In previous studies of frail elderly men and women, we have found that exercise training has numerous beneficial effects. We have found improvements in many measures of health and function in response to exercise, including aerobic exercise capacity, cardiovascular function, muscle strength, body composition, bone mineral density, blood pressure, glucose tolerance, and blood lipid profile. Moreover, weight loss may improve cardiovascular health risk factors, physical function, and quality of life in frail obese elderly subjects. There is also evidence that testosterone deficiency contributes to the age-related losses of muscle mass/and strength and BMD and increase fracture risk. Therefore, testosterone replacement therapy may help preserve muscle mass and BMD when obese older adults undergo lifestyle therapy with weight loss and exercise. In addition, subjects in the proposed research will receive several immediate benefits. The most important of these is repeated assessment and monitoring of several health factors including blood chemistries, body composition and bone density. The results of these assessments will be made available to the participants and their health care providers

Describe potential benefit(s) to society of the planned work.

Obesity in older adults, including many aging veterans, is a major public health problem. In fact, the public health success that has occurred in recent years could be in danger if lifestyles of older adults are neglected. The novel health outcomes and mechanistic-based data generated from this proposed RCT will have important ramifications for the standard of care for this rapidly increasing segment of the aging veteran population. The results could be incorporated in the MOVE (Managing Overweight/Obese Veterans) program, which currently does not have any guidelines for eligible veterans if they are 70 years or older.

Do anticipated benefits outweigh potential risks? Discuss the risk-to-benefit ratio.

The benefits to the subjects studied in this research protocol, and to society at large, far surpass the risks. As mentioned above, it is well known that exercise and weightl loss (lifestyle therapy) has multiple beneficial effects on health outcomes in obese adults. However, in frail elderly women and men, we also hypothesize that a multifactorial intervention by means of lifestyle therapy plus testosterone replacement will be the most effective approach for reversing sarcopenic obesity and frailty and thus further improving physical function, preserving muscle mass and quality, and bone density and quality. Among the side effects of testostesterone, erythrocytosis is a well recognized side effects but so long as patients are properly monitored and followed up will not result in signficant complications. While the relationshiop between testosterone and the risk for prostate cancer remains controversial, there is no data that supports a causal relationshipo between myocardial infaction and stroke. In addition, acne which has been reported as a side effect of tstosterone is not serious or life-threatening. In any event, results from this study would help define the optimal therapy to the public healthproblem of sarcopenic obesity and frailty, which might require a combination of lifestyle therapy and the restoration of the deficient

anabolic hormone (testosterone to normal levels) and thus potentially change the standard approach to obesity in older adults.

Section J: Consent Procedures

J1. Waiver of Consent

Will any portion of this research require a waiver of consent and authorization?

Yes

Please describe the portion of the research for which a waiver is required. (Example: chart review to determine subject eligibility)

Chart review to determine subject eligibility (for screening purposes only)

Explain why the research and the use or disclosure of protected health information involves no more than minimal risk (including privacy risks) to the individuals.

This waiver is needed to identify patients that may be eligible.

We will review the patients charts in the electronic medical record system in the VA known as Computerized Patient Record System (CPRS) in the same way as we review records of our clinical patients, the access to which is password protected. All members of the research team will be issued an individual password. We will also access CDW data and vital status files through the Data Access Request Tracker (DART) to include real SSNs in order to identify potential subjects for recruitment purposes.

We plan to protect idenfiers from improper use or disclosure.

For the CPRS records that are queried will be stored in folders on VA computer space to which access is restricted to personnel authorized by the VA Research and Development Committee (L drive), specifically the PI and study coordinator. Data will not be shared on hard drives of any workstation, or transmitted to any other facilty. All printing will be directed to a printer in a private office, not to common work areas. All data used will be kept until a schedule of destruction is approved.

For the CDW data and vital status files accessed through DART, the data will be transferred electronically to the local server (behind the VA firewall). Data access will be stored in a secure folder on the secure folder on Drive: L of the local VA server (behind the VA firewall). Data access will be restricted to the PI, programmer, and the project staff. Data will be used only to identify patients for study recruitment as outlined above. No data will be removed from this folder.

Explain why the waiver will not adversely affect the privacy rights and the welfare of the research subjects.

Data will be used only to identify patients for study recruitment. Any information that is collected as part of the research including PHI will not be used or disclosed to a third party except as required by law or permitted by a HIPAA authorization

Explain why the research could not practicably be conducted without the waiver and could not practicably be conducted without access to and use of the protected health information.

There are a large number of patients in the database and it is not possible to identify whether they might meet basic eligibility criteria without first accessing PHI and we would not be able to obtain HIPAA authorization from all of these patients prior

Describe how an adequate plan exists in order to protect identifiers from improper use and disclosure.

Access to the CPRS at the MEDVAMC is limited to individuals who are involved in patient care or human research and is password protected. Paper documents will be placed in double locked doors with only members of research team are allowed access. In addition, no names will be used in any communication or publication resulting from the study.

As also noted above, access to the CDW data and vital status files through DART, will be restricted to the PI, programmer, and the project staff. The data will be stored in a secure folder on Drive: L of the local VA server (behind the VA firewall). No data will be removed from this folder.

Describe how an adequate plan exists in order to destroy identifiers at the earliest opportunity consistent with conduct of the research, unless there is a health or research justification for retaining the identifiers or such retention is otherwise required by law.

Any files that contain identifiers for the specific purpose of screening and determining eligibility (as part of this waiver of consent) will be destroyed at the earliest opportunity (i.e. when no longer used to determine eligibility and recruit for the study) according to VA guidelines.

Describe how adequate written assurances exist in order to ensure that the PHI will not be reused or disclosed to (shared with) any other person or entity, except as required by law, for authorized oversight of the research study, or for other research for which the use or disclosure of the PHI would be permitted under the Privacy Rule.

Names will not be used in any communication or published reports about this study. In the event that samples had to be shared with other laboratories, coded samples will be transferred without identifiers.

The PHI will not be reused or disclosed to (shared with) any other person or entity, except as required by law, for authorized oversight of the research study, or for other research for which the use or disclosure of the PHI would be permitted under the Privacy Rule

Information from health records such as diagnoses, progress notes, medications, lab or radiology findings, etc.

Yes

Specific information concerning alcohol abuse:

No

Specific information concerning drug abuse:

No

Specific information concerning sickle cell anemia:

No

Specific information concerning HIV:

No

Specific information concerning psychiatry notes:

No

Demographic information (name, D.O.B., age, gender, race, etc.):

Yes

Full Social Security #:

Yes

Partial Social Security # (Last four digits):

Yes

Billing or financial records:

No

Photographs, videotapes, and/or audiotapes of you:

No

Other:

No

Will additional pertinent information be provided to subjects after participation?

No

If No, explain why providing subjects additional pertinent information after participation is not appropriate.

The information we review from the database to screen potential subjects are not new information (e.g. age, demographics) and/or are already part of their medical record system in the VA, for which they are getting ongoing medical care through their VA primary care providers. We are only reviewing medical records to determine whether or not potential subjects meet eligibility criteria and are not obtaining new tests or results during the medical record reviews.

J1a. Waiver of requirement for written documentation of Consent

Will this research require a waiver of the requirement for written documentation of informed consent?

No

J2. Consent Procedures

Who will recruit subjects for this study?

PI
PI's staff

Describe how research population will be identified, recruitment procedures, any waiting period between informing the prospective participant and obtaining consent, steps taken to minimize the possibility of coercion or undue influence and consent procedures in detail.

In order to determine whether or not potential subjects meet eligibility criteria, a review of medical records will be done and, for this purpose, a waiver of consent for the identification of potential subjects is attached in Section S. There are large

number of patients in the database and it is not possible to identify whether they might meet basic eligibility criteria without first accessing PHI. We will access the Corporate Data Warehouse (CDW) data and vital status files through the Data Access Request Tracker (DART) to include real SSNs in order to identify potential eligible subjects for recruitment purposes.

The CDW data are part of VA Informatics and Computing Infrastructure (VINCI), which is an initiative to improve researcher's access to VA data and to facilitate data analyses while ensuring Veteran's privacy and security. The data were collected for administrative/clinical reasons. Guidelines that allow data to be released as identified can be found in the DART user guide, which outlines an extensive review process of Project Documents and Approvals and Data Request Forms that undergo a privacy review and additional reviews by the Office of Research and Development and security review of information provided in the Research Request Memo. The patients will be recruited only for this research project.

We will recruit male veterans attending the Clinics at the MEDVAMC. Advertisements through posters and flyers, and direct mailings of printed materials to potential subjects will be done. Subjects will be identified from direct referral by the attending physician or by list generated from the Health Outcomes Section of patients who have low testosterone levels of <300 ng/dl and in the BMI and age range under the Inclusion Criteria. The records will be reviewed for exclusion criteria. A letter will be sent to the patient describing the study and introducing the investigators. Interested patients will be asked to return a postcard.

Individuals who express an interest in participation will undergo a brief telephone interview. Individuals who express an interest and meet the inclusion criteria will be invited to discuss their participation in greater detail. Detailed information will be provided regarding the aims of the study, and all of the tests that they will undergo if they participate. Verbal and written information about the potential benefits and risks of the study will be provided; their questions will be answered and any concerns will be addressed. If the individual is interested in participating, a screening evaluation will be scheduled. Prior to randomization, the volunteers will undergo a detailed medical history and physical examination, and a clinical laboratory. They will have the nature and purpose of the study explained to them again, discuss their reasons and motivation for participation to determine whether they are realistic and discuss any potential problems, that might interfere with participation and have their questions answered. Data which include HIPAA identifiers such as names, date of birth or age, dates of tests and medical record numbers (first letter of last name plus 4 digits of social security number) will be collected.

The consent will be obtained before any screening procedures included in the consent form are carried out.

Each subject will be informed that their participation in the study is completely voluntary and they may withdraw by telling the study team that they are no longer interested in participating in the study or they may send a withdrawal letter. They will also be informed that their choice will not at any time affect the commitment of their health care providers to administer care and that there will be no penalty or loss of benefits to which they are otherwise entitled.

Are foreign language consent forms required for this protocol?

No

J3. Privacy and Intrusiveness

Will the research involve observation or intrusion in situations where the subjects would normally have an expectation of privacy?

No

J4. Children

Will children be enrolled in the research?

No

J5. Neonates

Will non-viable neonates or neonates of uncertain viability be involved in research?

No

J6. Consent Capacity - Adults who lack capacity

Will Adult subjects who lack the capacity to give informed consent be enrolled in the research?

No

J7. Prisoners

Will Prisoners be enrolled in the research?

No

Section K: Research Related Health Information and Confidentiality

Will research data include identifiable subject information?

Yes

Information from health records such as diagnoses, progress notes, medications, lab or radiology findings, etc.
Yes

Specific information concerning alcohol abuse:
No

Specific information concerning drug abuse:
No

Specific information concerning sickle cell anemia:
No

Specific information concerning HIV:
No

Specific information concerning psychiatry notes:
No

Demographic information (name, D.O.B., age, gender, race, etc.):
Yes

Full Social Security #:
Yes

Partial Social Security # (Last four digits):
Yes

Billing or financial records:
No

Photographs, videotapes, and/or audiotapes of you:
No

Other:
No

At what institution will the physical research data be kept?
Paper records will be kept at Michael E DeBakey VA Medical Center, Building 110, Room 264 and Room 242.

How will such physical research data be secured?
Paper records will be kept at MEDVAMC Building 110, Room 236 and Room 242 in locked cabinets at all times and closely monitored by the research coordinator. Room 236 and Room 242 will also be locked every day and whenever the research coordinator steps out of the room. Therefore, the physical data will be secured by two locks. In addition, the entrance to Building 110 and entrance to our research laboratory (where Room 236 and Room 242 are located), is secured through PIV card access to approved research personnel only.

At what institution will the electronic research data be kept?
Data to include PHI will be stored at the Michael E DeBakey VA Medical Center located at S\Research\Villareal,Dennis\H-35267. Folder will be established by Rafael Garcia for PI.

Coded data (Study ID number) and PHI (limited to date of birth and date of visit, which are needed for specialized computer software to analyze the data) will also be entered, analyzed, and stored in specialized testing equipment (ParvoMed indirect calorimeter and Q-stress computer equipment in Room 254, Biodex dynamometer housed in Room 220 and 262, dual energy x-ray absorptiometer (DXA) and Peripheral CT scan in Room 226, Microindentation in Room 264, all located inthe PI's secured research lab at the second floor in Building 110, MEDVAMC and HR-peripheral CT scan in Room 140 in Building 109. All data will be coded, password protected, room locked protected, and entry to research lab will be PIV card-access protected (i.e. entry access to the research lab are given only to co-investigators using their PIV cards). Coded HR-pQCT data will be kept in Building 109, Room 138 in a computer locked, password protected. In addition, the entrance to Building 109 is secured through PIV card access to approved research personnel only. Also, Room 138 will be locked every day and whenever the research operator steps out of the room.

There will be software to be used for the conduct of the study, namely, Nutritional Data System for Research (Minneapolis, Minn) for analyses of food diaries, SF-36 software and Impact of Weight on Quality of Life software for analyses of Quality of Life, accelerometer software for analyses of physical activity, Hologic DXA for body comp analyses, Parvo for calorimetry, Biodex for strength, QCT software for bone density, Finite element analysis (FEA) software for bone strength assessment with HR-pQCT, Cardiac science for cardiac stress test, osteoprobe for microindentation, and Analyze/liceOmatic for MRI; nanodrop 200/200c (spectrophotometer for DNA, RNA quantification), SoftMax Pro 6.5.1 (ELISA plate reader); the PI has a licenses for these software's funded by his grant and SPSS and SAS statistical

software; for statistical analyses; the PI and VA have licenses for SAS software. Web application will NOT be used.

Coded data will also be stored in PI's Baylor computer network drive for analyses using specialized soft wares described above in PI's Baylor computer located in Building 110. Coded data are transferred to the PI's Baylor computer network drive using VA issued flash drive. Link to code are stored only in VA computer S drive. CD or DVD will also be used to back up data from the above medical device equipment and used to transfer the data to S drive. The CD or DVD are stored in locked cabinet and locked room (Room 226).

Flash drive will be used issued by the VA to the PI, which has VA FIPS 140-2 approved encryption. Data stored on mobile storage devices do not contain the only copy of research information. VA research data stored on all mobile devices or outside VA protected environment will be backed up regularly and stored securely within VA's protected environment.

Such electronic research data will be secured via BCM IT Services- provided secured network storage of electronic research data (Non-Portable devices only):

Yes

Such electronic research data will be secured via Other:

Yes, (describe below):
VA IT Services - provided secured network (Non-portable devices only)

Will there be anyone besides the PI, the study staff, the IRB and the sponsor, who will have access to identifiable research data?

Yes, identify the classes of the persons:
Federal agencies such as the Department of Veterans Affairs Office of Research Oversight, Office of Inspector General, Office of Human Research Protection, and Government Accounting Office and Food and Drug Administration (FDA) and Data and Safety Monitoring Board will be permitted to access patient records. Names will not be used in any published reports about the study. The research team may also share information with Hospital or University representatives, to complete Hospital or University responsiblities, primary care physician if a medical condition that needs uregent attention is discovered, and the Reserach Subjects Advocate at the VA. People who ensure the quality from the institutions where the research is being done, federal and other regulatory agencies will have access to all of the research data.

There is no plan to disclose or otherwise grant access to VINCI/CDW data to entities outside or within VHA other than described in this protocol. Only authorized personnel will have access to the data and personnel who no longer need the information will have their access removed.

Please describe the methods of transmission of any research data (including PHI, sensitive, and non-sensitive data) to sponsors and/or collaborators.

We will not transmit any PHI or sensitive data outside the MEDVAMC.

We will only send non-sensitive de-identified data to Dr. Clifford Qualls (Biostatistician who has a WOC VA appointment) via secure/encrypted VA email at the Biomedical Research Institute of New Mexico (the nonprofit organization associated with the New Mexico VA Health Care System) for statistical analyses. The data will be reviewed and confirmed as de-identified by the privacy officer before the de-identified data are sent to Dr. Clifford Qualls via secure/encrypted VA email.

Will you obtain a Certificate of Confidentiality for this study?

No

Please further discuss any potential confidentiality issues related to this study.

We will keep patient records according to approved Records Control Schedule for facility reserach data by the MEDVAMC.

It is understood by the PI that data will not be used or shared with others outside the scope of the reserach study as documented in the protocol approved by the IRB and MEDVAMC R and D Committee.

Removal of access to research study data will be accomplished for all study personnel when they are no longer part of the research team.

An Accounting of Disclosure (AOD) will be created and maintained for any disclosure of individually idnetifial ble information (III) outside the VA. The manual spreadsheet will include the data of the disclosure, nature or description of the III disclosed, purpose of each disclosure, and the ame and address of person or agency to which the disclosure was made.

Research records, including identifiers will be destroyed 6 years after cutoff (at the end of the fiscal year) after completion of the research project, but may be retained longer if required by other federal regulations or sponsor archive requirement

Section L: Cost/Payment

Delineate clinical procedures from research procedures. Will subject's insurance (or subject) be responsible for research related costs? If so state for which items subject's insurance (or subject) will be responsible (surgery, device, drugs, etc). If appropriate, discuss the availability of financial counseling.

Subjects will nor be responsible for any costs related to participation in the research. Any research-related costs will be borne by the research grant.

If subjects will be paid (money, gift certificates, coupons, etc.) to participate in this research project, please note the total dollar amount (or dollar value amount) and distribution plan (one payment, pro-rated payment, paid upon completion, etc) of the payment.

Dollar Amount:

250

Distribution Plan:

Participants will be compensated for their time and inconvenience: $50.00 after completing all the baseline testings and $ 50.00 after completing all the final testings (at 6 months). In addition, if they agree to the optional muscle studies, they will be paid $75.00 after each of the two outpatient muscle studies. This will take approximately (3-4) weeks after each completed visit to process.

Section M: Genetics

How would you classify your genetic study?

Discuss the potential for psychological, social, and/or physical harm subsequent to participation in this research. Please discuss, considering the following areas: risks to privacy, confidentiality, insurability, employability, immigration status, paternity status, educational opportunities, or social stigma.

Will subjects be offered any type of genetic education or counseling, and if so, who will provide the education or counseling and under what conditions will it be provided? If there is the possibility that a family's pedigree will be presented or published, please describe how you will protect family member's confidentiality?

Section N: Sample Collection

SAMPLE: Blood

What is the purpose of the sample collection?

Blood samples will be collected for 1) medical screening to determine eligility for the tests (see inclusion/exclusion criteria) 2) to monitor blood levels of testosterone, blood sugar, lipids, hormones, and markers of bone function and inflammation, and 3) for overall safety monitoring while in the study.

For blood draws, specify the amount drawn, in teaspoons, at each visit and across the course of the subjects entire participation time.

Up to a total of 4 tablespoons at baseline, and after 2 weeks, 3 months, and 6 months.

Is there the possibility that cell lines will be developed with this sample?No

Sample will be obtained from:

Clinical Labs, Research Labs

Will the sample be stripped of identifiers?

No

If sample will be released outside the hospital:

Will sample be released to anyone not listed as an investigator on the protocol? Will the information be identifiable, coded or de-identified?

Baylor C of Med, Wash U, U of Connect, U of Florida, U of Maryland, DE-IDENTIFIED for special tests

Will sample material be sold or transferred to any third parties? Will the information be de-identified?

No

If sample will be banked for future use:

Where will the sample be banked and for how long?

At the Michael DeBakey VA Medical Center for ten years or until used up. Specimens will have coded numbers and dates of tests.

Does the banking institution have an approved policy for the distribution of samples?

Yes

If the entire sample will NOT be used during the course of this research study:

Will the remaining tissue be discarded? If not what will be done with the remaining sample after study completion and how long will the sample be kept?

We will keep the samples for 10 years after study completion after which they will be discarded. We will request approval from IRB if we decide to reopen the study and reuse the samples for a new research protocol with related scope of interest.

Will samples be made available to the research subject (or his/her medical doctor) for other testing?

No

If a subject withdraws from the study:

Will subject have the option to get the remaining portion of their sample back?

No

Will samples be destroyed? If not, will they be kept anonymously? What will happen to the sample if the subject revokes authorization?

If the patient withdraws from the study, samples will not be destroyed and will be stored coded, except for the de identified samples that are sent to outside hospital for special tests mentioned above. If the subject revokes authorization, the samples will be destroyed or discarded, except for the de identified samples that are sent to outside hospital for special tests mentioned above.

Will data obtained from their sample be deleted? What will happen to the sample if the subject revokes authorization?

For those who withdraw from the study, we will use the data generated from the samples. However, for subjects who revoked authorization, their data will be deleted and samples destroyed or discarded.

Will study data or test results be recorded in the subject's medical records?

No

Will results of specific tests and/or results of the overall study be revealed to the research subject and or his/her doctor?

Standard test results that are done at VA lab and have clincal use are reported to their doctor (i.e. in CPRS, which is accessible to the vets primary care provider).

Any abnomal findings will be shared with the subject's primary care physician for further evalution and treatment through the CPRS which are shared with their VA primary care physicains. Depending on the urgency of the abnormal finidngs, the PI may directly contact the primary care physicain by phone to discuss the abnormal findings. The PHI that may be used or disclosed to the subjects primary care physician may include names, date of bith or age, and date of tests.

Please identify all third parties, including the subject's physician, to receive the test results.

Subject's treating physician

<u>SAMPLE: Tissue</u>

What is the purpose of the sample collection?

We will collect muscle samples to address secondary aim: "determine the effect of lifestyle therapy plus testosterone compared to lifestyle therapy plus placebo on catabolic and anabolic factors in skeletal muscles". We will compare changes in cellular/molecular, gene, and protein expression of skeletal muscle factors invoved in inflammation, anabolism, and catabolism.

For blood draws, specify the amount drawn, in teaspoons, at each visit and across the course of the subjects entire participation time.

Is there the possibility that cell lines will be developed with this sample?No

Sample will be obtained from:

Clinical Labs, Research Labs

Will the sample be stripped of identifiers?

No

If sample will be released outside the hospital:

Will sample be released to anyone not listed as an investigator on the protocol? Will the information be identifiable, coded or de-identified?

Yes, to potential collaborators for specialized tests or equipment. Samples will be coded.

Will sample material be sold or transferred to any third parties? Will the information be de-identified?

No,

If sample will be banked for future use:

Where will the sample be banked and for how long?

Michael De Bakey VA Medical Center for up to 10 years after study completion.

Does the banking institution have an approved policy for the distribution of samples?

Yes

If the entire sample will NOT be used during the course of this research study:

Will the remaining tissue be discarded? If not what will be done with the remaining sample after study completion and how long will the sample be kept?

The remaining samples will be stored for up to 10 years after study completion.

Will samples be made available to the research subject (or his/her medical doctor) for other testing?

No

If a subject withdraws from the study:

Will subject have the option to get the remaining portion of their sample back?

No

Will samples be destroyed? If not, will they be kept anonymously? What will happen to the sample if the subject revokes authorization?

For patients who withdraw from the study, the samples will not be destroyed but will be stored anonymously. For those who revoke authorization, samples will be destroyed or discarded.

Will data obtained from their sample be deleted? What will happen to the sample if the subject revokes authorization?

For those who withdraw from the study, we will use the data generated from the samples. However, for patients who revoked authorization, their data will be deleted.

Will study data or test results be recorded in the subject's medical records?

Yes

Will results of specific tests and/or results of the overall study be revealed to the research subject and or his/her doctor?

No

Please identify all third parties, including the subject's physician, to receive the test results.

None

Section O: Drug Studies

Does the research involve the use of ANY drug* or biologic? (*A drug is defined as any substance that is used to elicit a pharmacologic or physiologic response whether it is for treatment or diagnostic purposes)

Yes

Does the research involve the use of ANY gene transfer agent for human gene transfer research?

No

O1. Current Drugs

Drug : testosterone

Is this study placebo-controlled?

Yes

If yes, be sure that you justify the use of the placebo for this research in the space below.

All participants are obese and all will get the benefits of weight loss and exercise therapy (lifestyle therapy) to improve overall health and physical function. However, it is not known whether adding testosterone replacement to lifestyle therapy in obese older pateints will further improve overall health and physical function and importantly preserve muscle and bone mass while undergoing weight loss. The use of the placebo is necessary in order to conduct a randomized, comparative-efficacy, double-blind, placebo controlled (for testosterone) trial of the effects of lifestyle therapy + testosterone replacement versus lifestyle therapy + placebo and provide high-level evidence to support the hypothesis that a multifactorial intervention by means of lifestyle therapy + testosterone replacement will be the most effective approach for reversing sarcopenic obesity and frailty in obese older veterans, and, thus this high level of evidenced-based data could be used to change the standard treatment approach for obese older adults and incorporated into the MOVE (Managing Overweight/Obese Veterans) program.

Will the research involve a radioactive drug?

No

Section P: Device Studies

Does this research study involve the use of ANY device?

No

Section Q. Consent Form(s)

None

Section R: Advertisements

Mode of Advertising: Bulletin Board

Exact language of Advertisement:

OLDER VETERAN VOLUNTEERS NEEDED

To participate in a study looking into the effect of testosterone, weight loss, and exercise training on physical function, body fat, muscle and bone.

Duration: Participation in the study will be ~ 8 months.

Tests/Procedures include medical examination, assessment of physical function, cardiovascular testing, blood tests, body composition testing, mood/cognition, and muscle biopsies

Interventions: Supervised weight loss and exercise training program ± testosterone therapy

Compensation: may receive up to $ 250.00

Dr. Dennis Villareal Principal Investigator HRRC No. H35267 Please contact the study office at telephone no. (713) 578-4300 for more information
